# Phallusiasterols A and B: Two New Sulfated Sterols from the Mediterranean Tunicate *Phallusia fumigata* and Their Effects as Modulators of the PXR Receptor

**DOI:** 10.3390/md12042066

**Published:** 2014-04-03

**Authors:** Concetta Imperatore, Filomena D’Aniello, Anna Aiello, Stefano Fiorucci, Claudio D’Amore, Valentina Sepe, Marialuisa Menna

**Affiliations:** 1 The NeaNat Group, Department of Pharmacy, University of Naples “Federico II”, Via D. Montesano 49, Napoli 80131, Italy; E-Mails: cimperat@unina.it (C.I.); filomena.daniello@unina.it (F.D.); aiello@unina.it (A.A.); 2 Department of Clinical and Experimental Medicine, Faculty of Medicine, University of Perugia, Via Gerardo Dottori 1, S. Andrea delle Fratte, Perugia 06132, Italy; E-Mails: fiorucci@unipg.it (S.F.); claudiodamore1983@gmail.com (C.D.); 3 Department of Pharmacy, University of Naples “Federico II”, Via D. Montesano 49, Napoli 80131, Italy; E-Mail: valentina.sepe@unina.it

**Keywords:** sterols, ascidians, tunicates, *Phallusia fumigata*, NMR, PXR receptor

## Abstract

Purification of the apolar extracts of the marine ascidian *Phallusia fumigata*, afforded two new sulfated sterols, phallusiasterols A (**1**) and B (**2**). The structures of the new compounds have been elucidated using mass spectrometry and NMR experiments. The effects of phallusiasterols A and B as modulators of pregnane-X-receptor (PXR) have been investigated. These studies revealed that phallusiasterol A induces PXR transactivation in HepG2 cells and stimulates the expression of the PXR target genes CYP3A4 and MDR1 in the same cell line. Molecular docking calculations suggested the theoretical binding mode of phallusiasterol A with hPXR and revealed that phallusiasterol A fitted well in the LBD of PXR.

## 1. Introduction

Marine invertebrates are a prolific source of unconventional steroids. More than 1600 new steroidal structures have been so far isolated [1,2] with structural modifications including oxygenation, alkylation, esterification, and sulfation of both the nucleus and the side chain, extensive modification of the latter, or bond cleavage in the rings of the tetracyclic nucleus leading to degradation of the conventional carbon backbone [3,4,5,6]. Steroids of marine origin have exhibited a diverse array of pharmacological activities, such as antimicrobial, cytotoxic, antifouling, ichthyotoxic, and antiinflammatory [1,2,3,4]. Nevertheless, the sterol composition of marine ascidians has received much less attention than those of other invertebrates. It has been shown that, in general, tunicates contain ∆^5^ sterols bearing conventional side chains with cholesterol as the major component and cholestanol and cholest-7-en-3β-ol as the minor ones [1]. Among the minor unconventional sterols isolated from tunicates, 5,8-endoperoxides from several ∆^5,7,9(11)^ sterols [7] together with 24-hydroperoxy-24-vinylcholesterol and the corresponding 24-hydroxy derivative from *Phallusia mamillata* and *Ciona intestinalis* [8], ∆^4^-3-keto steroids, 5β-stanols, and 4-methyl sterols in addition to endoperoxides from *Ascidia nigra* [9,10], two short side chain sterols (C-22 and C-23) from *Polizoa opuntia* [11], and four 9,11-secosterols from *Aplidium conicum* [12] have been reported. These findings, although few number, show that ascidians can produce sterols with unique structural features and they could be a good source of interesting novel compound.

As a part of our research program aimed to discover new bioactive metabolites from marine tunicates, we have investigated the Mediterranean ascidian *Phallusia fumigata*. This study led to the isolation of two new sulfated sterols phallusiasterols A and B (**1** and **2**, Figure 1 and Supplementary Figures S1–S23), which, to our knowledge, represent the first example of sulfated sterols isolated from tunicates. Based on the reported activity of sulfated marine sponge steroids as nuclear receptor ligands, the effects of phallusiasterols A and B as modulators of pregnane X receptor (PXR) have been investigated *in vitro*. These studies revealed that phallusiasterol A induces PXR transactivation in HepG2 cells and stimulates the expression of the PXR target genes CYP3A4 and MDR1 in the same cell line.

**Figure 1:**
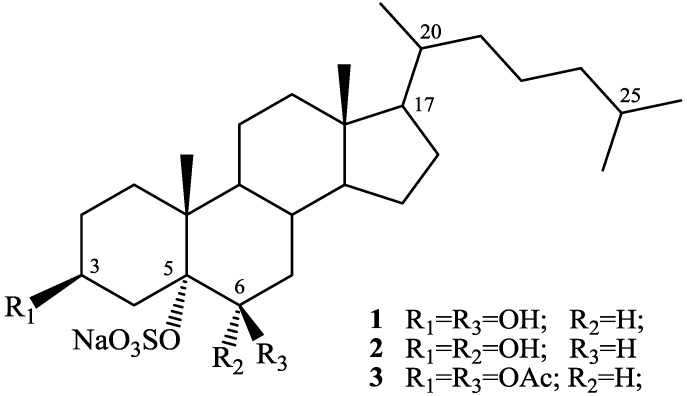
Structures of phallusiasterols A (**1**) and B (**2**).

PXR is a master gene orchestrating the expression of a wide family of genes involved in uptake, metabolism, and disposal of a number of endo- and xenobiotics, including drugs, bile acids, steroid hormones, and metabolic intermediates in mammalian cells. PXR is almost exclusively expressed in the gastrointestinal tract and liver, with lower levels in the kidney and ovary. PXR dysfunction is associated with immune disorders and inflammatory bowel diseases, including ulcerative colitis and Crohn’s disease. Chemical and pharmacological characterization of marine steroid libraries has allowed the identification of a number of selective PXR agonists (natural and synthetic compounds) which have been effective in reducing nuclear factor (NF)-κB activity and intestinal inflammation. These findings open the possibility of discovering potential leads for the treatment of liver and intestinal disorders [13].

## 2. Results and Discussion

### 2.1. Isolation and Structure Elucidation

A series of subsequent normal-phase chromatographies of the ethyl acetate extract of the ascidian *P. fumigata* collected from the bay of Pozzuoli (Napoli, Italy), allowed the isolation of compounds **1** and **2** (Figure 1) in pure form.

The high field region of the ^1^H-NMR spectrum (pyridine-*d*_5_) of phallusiasterol A (**1**) contained signals for five methyl groups of a steroidal nucleus: two singlets a δ 0.67 (Me-18) and 1.60 (Me-19) and doublets at δ 0.96 (*J* = 6.5 Hz, H_3_-21), 0.89 and 0.88 (*J* = 6.6 Hz, H_3_-26 and H_3_-27). A pseudomolecular ion at *m*/*z* 545.2858 [M + Na]^+^ was observed in the high-resolution ESI mass spectrum (positive ion mode), indicating for **1** a molecular formula of C_27_H_47_SO_6_Na (calcd. 545.2889). The MS/MS fragmentation pattern of **1** was compatible with the presence of a sulfate group, displaying the peak at *m*/*z* 425.3392 [M − NaHSO_4_ + Na]^+^. Consistent with the MS data, the ^1^H and ^13^C-NMR spectra of **1** contained two hydroxymethine signals [δ_H_ 4.33, bs, δ_C_ 75.3 (CH); δ_H_ 4.79, m, δ_C_ 67.2 (CH)] and a highly deshielded unprotonated carbon (δ 87.8), presumably the locus of the sulfate group. This assumption was verified by acetylation of phallusiasterol A (**1**) which gave the corresponding diacetate **3** (Figure 1 and Supplementary Figure S24), thus confirming, according to MS information, the presence of two secondary alcohols and a quaternary sulfoxy group in **1**.

**Figure 2:**
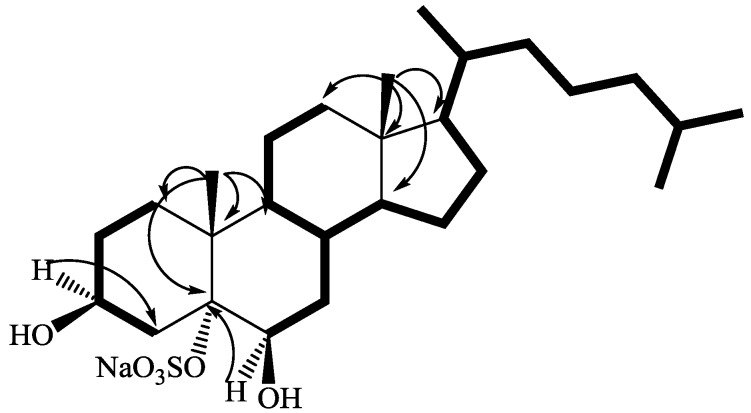
COSY connectivities (bold bonds) and selected HMBC (from H to C) correlations of **1**.

The whole series of 2D NMR data allowed us to locate the hydroxyl groups at C-3, C-6, and the sulfate group at C-5. Analysis of COSY spectrum of **1** (pyridine-*d*_5_), assisted by TOCSY information, allowed the sequential assignment of all the protons of the tetracyclic system (Figure 2) whereas the protonated ^13^C signals were assigned to the relevant protons from HSQC data (Table 1).

The steroidal skeleton of **1** was assembled on the base of key HMBC correlations of H_3_-19 with C-1, C-5, C-9, and C-10 and of H_3_-18 with C-12, C-13, C-14, and C-17 (Figure 2). Information on the side chain were also provided by analysis of 2D NMR data; in the COSY spectrum, the spin system from H-6 to H-17 (through H-14) was extended to the side chain protons through the correlation between H-17 (δ 1.10) and the multiplet at δ 1.36 (H-20), which is in turn coupled to both the methyl doublet at δ 0.96 (H_3_-21) and the methilene protons at δ 1.38 and 1.03 (H-22a and H-22b). The sequence was extended to the H_3_-26 and 27 methyls, taking also advantage of HSQC and TOCSY information. 

**Figure 3:**
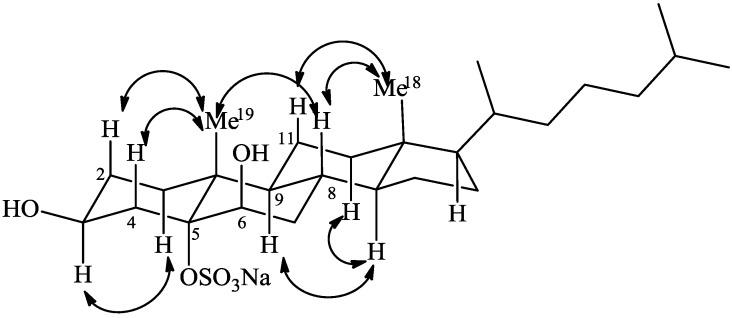
Key ROESY correlations detected for **1**.

The relative stereochemistry of phallusiasterol A (**1**) was established through analysis of ROESY data (Figure 3) and consideration of both coupling constants and solvent shifts (∆δ = δ CDCl_3_ − δ pyridine-*d*_5_) observed for some key signals in the ^1^H-NMR spectrum of **1**. The equatorial orientation of the hydroxyl group at C-3, assigned to the β face, was easily deduced by the presence of an axial-axial coupling constant (*J* = 13.1 Hz) found for the vicinal H-4_β_. Furthermore, a large pyridine-induced downfield shift (−0.46 ppm) was observed for the H-3_α_ signal, which resonates at δ 4.33 when the proton spectrum is recorded in CDCl_3_ (see Experimental Section). This indicated a 1,3-diaxial interaction of H-3_α_ proton with the polar sulfate group linked at C-5, which therefore must be α-oriented [14,15,16]. The axial orientation of H-8, H-9, and H-14 was apparent from the coupling constants values of these protons (Table 1). These data, combined to the observation of ROESY correlations of H_3_-19 to H-4_β_, to H-2_β_, and to H-8, of H_3_-18 to H-8, and of H-9 to H-14 (Figure 3), defined the A/B, B/C, and C/D *trans* ring junctions of the 5α-cholestane nucleus of **1**.

The small coupling constants showed by H-6 (δ 4.33, bs) suggested a β-orientation of the OH group at C-6. This was confirmed by the signal large pyridine-induced downfield shift observed for H-4_β_ (−0.75 ppm). According to Fujimoto *et al.* [17], the resonances of H-4 protons in 3β,5α,6-cholestanetriols show a diagnostic dependence on the configurations at C-6. Their resonances are strongly influenced by a deshielding effect of the 6-OH group through a 1,3-diaxial (with the 6β isomer) or 1,3-diequatorial (with the 6α isomer) interaction, which is intensified in pyridine solution. Further significant pyridine-induced shifts were observed for H_3_-19 (−0.32 ppm) and H-8 (−0.31 ppm), which supported the axial orientation of the hydroxyl group at C-6. The orientation of substituents at C-17 and C-20 in phallusiasterol A, as shown in Figure 1, was assumed to be the same as in related polyhydroxysterols due to the almost identical values of carbon chemical shifts around these carbon atoms [16,17,18]. The structure of phallusiasterol A was hence established as 3β,6β-dihydroxy-5α-cholestan-5α-yl sodium sulfate. 

**Table 1:** ^1^H (700 MHz) and ^13^C (125 MHz) NMR data for phallusiasterols A and B in pyridine-*d*_5_.

	Phallusiasterol A (1)			Phallusiasterol B (2)	
Pos.	δ_C_	δ_H_ (mult., *J* in Hz)	HMBC	δ_C_	δ_H_ (mult., *J* in Hz)
1α/ax	34.4	1.90 (dt,13.4, 4.2)	2, 5, 10, 19	33.8	2.10, m
1β/eq		1.56, m	2, 3, 5, 10, 19		1.50, m
2α/eq	31.8	2.20, m	1, 3, 10	32.5	2.22, m
2β/ax		2.01 ^a^	1, 3, 9		1.97, m
3α/ax	67.2	4.79, m	1, 2, 4	67.4	4.72, m
4α/eq	43.2	2.51 (dd, 13.6, 4.5)	2, 3, 5, 6, 10	43.3	2.30 (dd, 13.0, 4.0)
4β/ax		3.10, m	2, 3		2.81, m
5	87.8	-	-	75.8	-
6α	75.3	4.33 (bs)	4, 5, 8, 10	66.1	4.34, (d, 5.5)
7α/ax	35.1	1.88 ^a^	5, 6, 8, 9	35.7	1.93, m
7β/eq		2.21, m	8, 14		2.45, m
8β/ax	31.1	2.05 (qd, 11.6, 4.3)	7, 9, 14	30.9	1.99 (qd, 11.0, 3.4)
9α/ax	46.9	1.75, (ddd, 13.6, 11.1, 3.6)	8, 10, 11, 19	45.5	1.88 (ddd, 13.5, 11.2, 3.6)
10	40.6	-	-	39.8	-
11α/eq	21.8	1.47 (dq, 14.1, 3.8)	9, 10, 12	21.7	1.48, m
11β/ax		1.37 ^a^	9, 12, 17		1.38 ^a^
12α/ax	40.2	1.13, m	11, 14	40.8	1.17 ^a^
12β/eq		1.95 ^a^	9, 13, 14		1.94 (dt, 12.4, 3.4)
13	42.9	-	-	43.4	-
14α	56.2	1.05, m	8, 13, 15, 16, 18	56.1	1.02, m
15α	24.4	1.57 ^a^	13, 14, 16, 17	24.4	1.55, m
15β		1.04, m	8, 14, 16		1.07, m
16α	28.5	1.82 (ddd, 13.6, 9.5, 3.7)	13, 15, 17	29.1	1.83 (ddd, 13.6, 9.4, 3.8)
16β		1.21 ^a^	13, 17, 20		1.23, m
17	56.4	1.10, m	13, 15, 16, 20, 22	56.9	1.11, m
18	12.4	0.67, s	12, 13, 14, 17	12.3	0.71, s
19	18.7	1.60, s	1, 5, 9, 10	18.5	1.47, s
20	36	1.36, m	17, 21, 22, 23	36.8	1.35, m
21	19	0.96 (d, 6.5)	17, 20, 22	19	0.96 (d, 6.5)
22a	36.5	1.38 ^a^	20, 21, 24	36.5	1.37 ^a^
22b		1.03, m	20, 21, 24		1.01, m
23a	24.2	1.38 ^a^	24	24.1	1.36 ^a^
23b		1.18 ^a^	24		1.17, m
24a	39.7	1.13, m	23, 26, 27	39.7	1.14, m
24b		1.13, m	23, 26, 27		1.14, m
25	28.3	1.51, m	23, 24, 26, 27	28.7	1.52 ^a^
26	22.7	0.88 (d, 6.6)	24, 25	22.8	0.88 (d, 6.5)
27	22.9	0.89 (d, 6.6)	24, 25	22.9	0.88 (d, 6.5)

^a^ Overlapped by other signals.

The molecular formula C_27_H_47_SO_6_Na established for the second metabolite phallusiasterol B (**2**) by HRESIMS and NMR data was identical to that of **1**, indicating that the compounds were isomers. The ^1^H and ^13^C-NMR resonances of **2** closely resembled those of **1**, except for some signals surrounding C-6 (Table 1). Interpretation of COSY, TOCSY, HSQC and HMBC 2D NMR experiments provided evidence for the same planar structure for **2** as that of **1**. The difference between **1** and **2** was traced to a different stereochemistry at C-6, with the OH group α oriented in compound **2**. This was deduced from the different shape of H-6 signal in **2** (δ 4.34, d, *J* = 5.5 Hz) when compared to that of H-6 in **1** (δ 4.33, bs). The upfield shift of C-6 in the ^13^C-NMR spectrum of **2** (δ 66.1) relative to that observed for **1** (δ 75.3) added support to this assignment [18,19,20]. Conclusive evidence was achieved from ROESY spectrum; a strong correlation was observed between H-6 and H_3_-19 and the β orientation of H-6 is the only position according with this demand. In addition, on comparison of the proton spectra of **2** recorded in CDCl_3_ and pyridine-*d*_5_, a pyridine-induced deshielding was observed for H-4_α_ (−0.64 ppm), comparable to that observed for H-4_β_ in **1**, indicating a 1,3-diequatorial interaction of this proton with the 6α OH group [17]. Thus, phallusiasterol B was identified as the epimer at C-6 of phallusiasterol A.

### 2.2. Biological Evaluation

**Figure 4:**
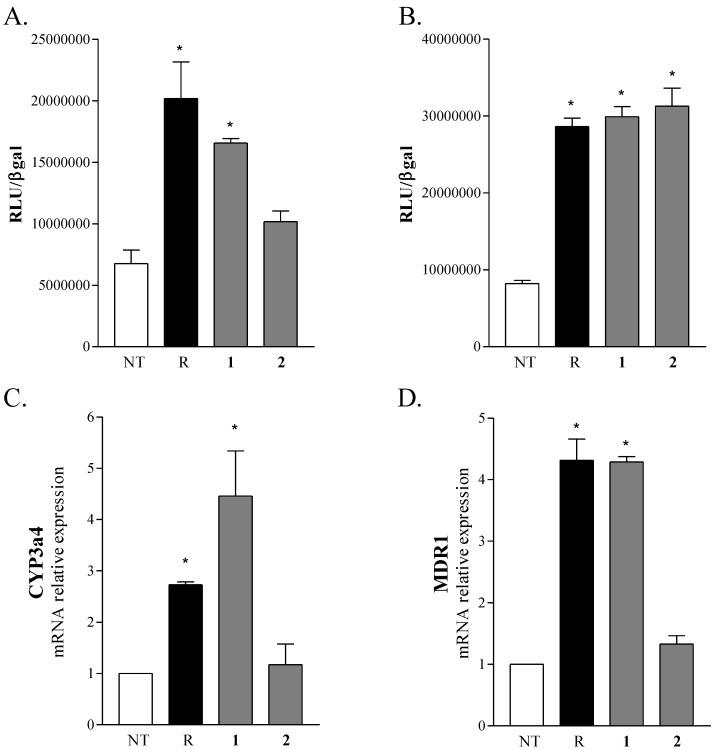
(**A**,**B**) Luciferase reporter assay. HepG2 cells were transiently transfected with pSG5-pregnane-X-receptor (PXR), pSG5-RXR, pCMV-βgalactosidase and p(CYP3A4)-TK-Luc vectors and then stimulated with (A) 10 μM rifaximin, phallusiasterol A (**1**) or phallusiasterol B (**2**) for 18 h, or (B) 10 μM rifaximin alone or in combination with 50 μM of compounds **1** or **2**. Relative Luciferase Units were normalized with β-galactosidase Units (RLU/βgal). (**C**,**D**) Real-Time PCR analysis of CYP3A4 and MDR1 expression in HepG2 cells primed with 10 μM rifaximin, compounds **1** or **2** for 18 h. Values were normalized relative to GAPDH mRNA and expressed relative to those of not treated cells, which were arbitrarily set to 1. All experiments were performed in triplicate. NT, not treated cells. R, Rifaximin. * *P* < 0.05 *versus* NT cells. Data are mean ± SE.

The role of marine steroids as nuclear receptor ligands has been recently highlighted [13] and several sulfated marine steroids have been identified as a new class of the pregnane X receptor (PXR) agonists [21,22]. Based on this background, we have investigated a possible role of phallusiasterols A (**1)** and B (**2**) in regulating the PXR activity. A transactivation assay on HepG2 cells, a human hepatocarcinoma cell line, has been performed, as described in the Experimental Part. As shown in Figure 4A, only compound **1** acted as PXR agonist at concentration of 10 μM; its activity was comparable to that of rifaximin, a well characterized ligand for the human PXR. As shown in Figure 4B, both compounds **1** and **2** failed to reverse the induction of luciferase activity caused by rifaximin, indicating that they were not PXR antagonists. Similar results have been obtained by analyzing the effect exerted by **1** and **2** in terms of regulation of PXR mediated induction of two PXR target genes, CYP3A4 (Figure 4C) and MDR1 (Figure 4D), in the same cell line. Compound **1** effectively stimulated the expression of both target genes, whereas **2** failed to induce them. These results have an important implication in terms of structure-activity relationship, because they highlight a crucial role in the ligand-receptor binding of phallusiasterols of the configuration at C-6.

### 2.3. Docking Studies

We then analyzed by means of molecular docking calculations the interactions of phallusiasterol A with hPXR. The calculations were run by Autodock4.2 software [23]. The hPXR presents a large ligand binding cavity, allowing the accommodation of both small and large ligands and the number of chemicals has grown rapidly, including many drugs in use such as statins, antibiotic rifampicin and its derivative rifaxim, antihypertensive drugs nifedipine, as well as pesticides, environmental toxicant, plasticizers.

**Figure 5:**
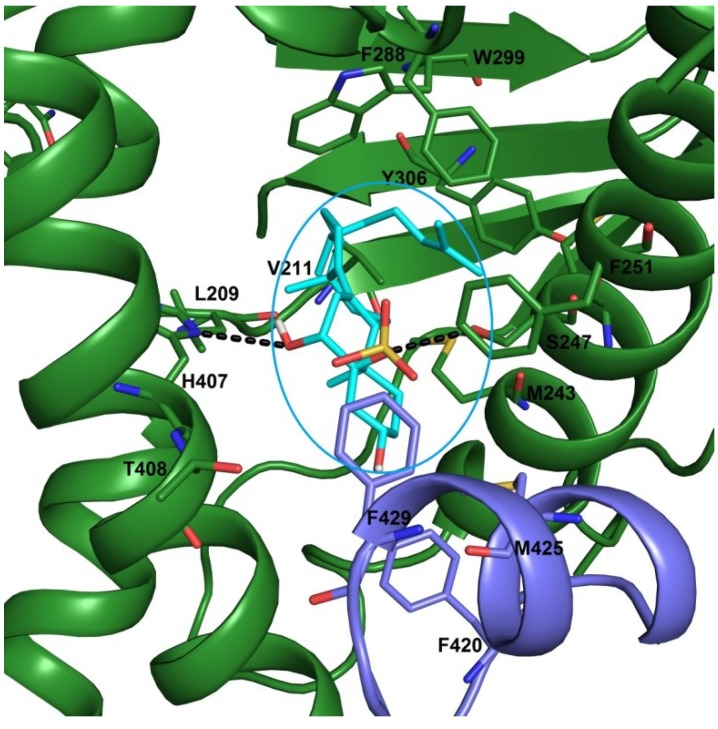
Binding mode of phallusiasterol A **1** (cyan sticks), predicted by docking calculations in the PXR LBD (PBD code 3HVL). PXR is shown as green cartoon, AF-2 helix is colored in violet. Amino acids involved in ligand binding are shown as green and violet sticks. All hydrogen atoms are omitted for clarity.

In a previous work [21], the possible interactions of solomonsterols A and B with hPXR has been reported. In this model, the 2-O and 3-O sulfate groups exert hydrogen bonds with His407 and Ser247, respectively. In this study three different x-ray structures of the PXR LBD (pdb codes: 3hvl, 1nrl and 1m13) have been used. As shown in Figure 5, the OH at C-6 and the 5-O sulphate groups form hydrogen bonds with NH of His407 and OH of Ser247, respectively. The steroidal scaffold engages Van der Waals interactions with hydrophobic residues of LBD, such as Leu209, Met243 and Phe251, and the flexible side chain is settled in a hydrophobic pocket establishing several favourable contacts with Met250, Phe288, Trp299 and Tyr306 (Figure 5). Noteworthy, in this pose, phallusiasterol A is oriented to form further hydrophobic interactions with Phe420 and Met425. These last residues are on a flexible α-helix (AF-2 helix) in the activation function 2-region (AF-2). This part of receptor is responsible for binding of the co-activator or co-repressor peptides. In conclusion, the present docking analysis revealed that phallusiasterol A fitted well in the LBD of PXR and could be stabilized PXR in agonist conformation with consequent conformational change and co-activator recruitment.

## 3. Experimental Section

### 3.1. General Experimental Procedures

High-resolution ESI-MS spectra were performed on a Thermo LTQ Orbitrap XL mass spectrometer. The spectra were recorded by infusion into the ESI source using MeOH as the solvent. Optical rotations were measured at 589 nm on a Jasco P-2000 polarimeter using a 10-cm microcell. NMR spectra were determined on Varian Unity Inova spectrometers at 700 and 500 MHz; chemical shifts were referenced to the residual solvent signal (C_5_D_5_N: δ_H_ 8.71, 7.55, 7.19, δ_C_ 149.9, 135.5, 123.5; CDCl_3_: δ_H_ 7.26, δ_C_ 77.0; C_6_D_6_: δ_H_ 7.15, δ_C_ 128.0). For an accurate measurement of the coupling constants, the one-dimensional 1H-NMR spectra were transformed at 64K points (digital resolution: 0.09 Hz). Homonuclear ^1^H connectivities were determined by COSY experiment. Through-space ^1^H connectivities were evidenced using a ROESY experiment with a mixing time of 500 ms. Two and three bond ^1^H–^13^C connectivities were determined by gradient 2D HMBC experiments optimized for a ^2,3^*J* of 8 Hz. ^3^*J*_H–H_ values were extracted from 1D ^1^H-NMR. High performance liquid chromatography (HPLC) separations were achieved on a Shimadzu LC-10AT apparatus equipped with a Knauer K-2301 refractive index detector.

### 3.2. Collection, Extraction, and Isolation

Specimens of *Phallusia fumigata* were collected in April 2008 at in the bay of Pozzuoli (Napoli, Italy). The samples were frozen immediately after collection and stored at −20 °C until extraction. A reference specimen is deposited at the Dipartimento di Farmacia, University of Naples. The fresh thawed animals (424 g of dry weight after extraction) were homogenized and extracted twice with methanol and then twice with chloroform (4 × 200 mL). The combined extracts were concentrated in vacuo, and the resulting aqueous residue was extracted with EtOAc and subsequently with *n*-BuOH. Separation of the EtOAc soluble material (1.04 g) was achieved by gradient silica gel MPLC (hexane→EtOAc→MeOH). The fraction eluted with hexane/EtOAc 3:7 v/v, (37.0 mg) was chromatographed by HPLC on a SiO_2_ column (Luna 5 μm, 250 × 4.60 mm) eluting with hexane/EtOAc 55:45 (v/v), yielding a fraction (3.3 mg) which has been further purified by HPLC on a SiO_2_ column (Luna 5 μm, 250 × 4.60 mm), eluting with hexane/propan-2-ol 93:7, thus affording phallusiasterol A (2.0 mg) and B (1.1 mg) as pure compounds.

### 3.3. Phallusiasterol A (**1**)

Colorless amorphous solid, 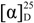 −3.5 (*c* 0.1, CHCl_3_); HRESIMS (positive ion mode, CH_3_OH) *m*/*z* 545.2858 ([M + Na]^+^, calcd. for C_27_H_47_SO_6_Na_2_^+^ 545.2889); ^1^H and ^13^C-NMR (C_5_D_5_N): see Table 1. ^1^H-NMR (CDCl_3_): δ 4.33 (1H, m, H-3), 3.96 (1H, br s, H-6), 2.34 (1H, dd, *J* = 13.1, 10.5 Hz, H-4β), 2.02 (1H, m, H-7β), 1.99 (1H, overlapping, H-4α), 1.98 (1H, overlapping, H-12β), 1.90 (1H, m, H-2α), 1.83 (1H, ddd, *J* = 13.6, 9.5, 3.7, H-16α), 1.74 (1H, qd, *J* = 12.0, 4.3 Hz, H-8β), 1.68 (1H, dt, *J =* 13.4, 4.2 Hz, H-1α), 1.60 (1H, overlapping, H-7α), 1.57 (1H, overlapping, H-2β), 1.57 (1H, overlapping, H-15α), 1.54 (1H, m, H-9α), 1.51 (1H, m, H-25), 1.48 (1H, ddd, *J* = 13.4, 4.5, 2.3, H-1β), 1.41 (1H, dq, *J* = 14.1, 3.8, H-11α), 1.37 (1H, m, H-20), 1.35–1.33 (2H, overlapping, H-22a and H-23a), 1.29 (1H, overlapping, H-11β), 1.28 (3H, s, Me-19), 1.26 (1H, overlapping, H-16β), 1.25 (1H, m, H-14α), 1.24 (1H, overlapping, H-23b), 1.18 (1H, m, H-12α), 1.15–1.10 (3H, m, H-17, H-24a, and H-24b), 1.07 (1H, m, H-15β), 1.00 (1H, m, H-22b), 0.91 (3H, d, *J* = 6.5 Hz, Me-21), 0.87 (3H, d, *J* = 6.6 Hz, Me-26), 0.86 (3H, d, *J* = 6.6 Hz, Me-27), 0.67 (3H, s, Me-18). ^13^C-NMR (CDCl_3_): δ 84.2 (C-5), 75.8 (C-6), 67.8 (C-3), 56.0 (C-17), 55.8 (C-14), 46.0 (C-9), 42.8 (C-13), 41.3 (C-4), 39.8 (C-10 and C-12), 39.6 (C-24), 36.2 (C-22), 35.8 (C-20), 34.0 (C-7), 33.6 (C-1), 30.5 (C-2), 30.4 (C-8), 28.2 (C-16), 28.0 (C-25), 24.0 (C-15), 23.8 (C-23), 21.3 (C-11), 22.7 (Me-26), 22.5 (Me-27), 18.8 (Me-21), 18.2 (Me-19), 12.1 (Me-18).

### 3.4. Phallusiasterol B (**2**)

Colorless amorphous solid, 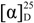 +7.9 (*c* 0.1, CHCl_3_); HRESIMS (positive ion mode, CH_3_OH) *m*/*z* 545.2870 ([M + Na]^+^, calcd. for C_27_H_47_SO_6_Na_2_^+^ 545.2889); ^1^H and ^13^C-NMR (C_5_D_5_N): see Table 1. ^1^H-NMR (CDCl_3_): δ 4.05 (1H, m, H-3), 3.84 (1H, d, *J* = 5.4, H-6), 2.25 (1H, t, *J* = 13.2, H-4β), 1.99 (1H, dt, *J* = 12.4, 3.4, H-12β), 1.96 (1H, m, H-7β), 1.67 (1H, dd, *J* = 13.2, 2.1, H-4α), 1.87–1.84 (3H, overlapping, H-2α, H-7α, H-8β H-16α), 1.68 (1H, dd, *J* = 13.1, 4.7, H-9α), 1.56-1.54 (3H, overlapping, H-1α, H-2β, and H-15α), 1.51 (1H, m, H-25), 1.42–1.41 (2H, m, H-1β and H-11α), 1.37 (1H, m, H-20), 1.33 (2H, overlapping, H-22a and H-23a), 1.30 (1H, overlapping, H-11β), 1.28 (3H, s, Me-19), 1.27-1.24 (3H, overlapping, H-14α, H-16β, and H-23b), 1.12 (1H, m, H-12α), 1.20-1.11 (3H, m, H-17, H-24a, and H-24b), 1.07 (1H, m, H-15β), 1.00 (1H, m, H-22b), 0.91 (3H, d, *J* = 6.5 Hz, Me-21), 0.87 (3H, d, *J* = 6.6 Hz, Me-26), 0.86 (3H, d, *J* = 6.6 Hz, Me-27), 0.70 (3H, s, Me-18). ^13^C-NMR (CDCl_3_): δ 77.2 (C-5), 67.3 (C-3), 63.5 (C-6), 56.4 (C-17), 55.6 (C-14), 46.1 (C-9), 42.9 (C-13), 41.6 (C-4), 40.2 (C-12), 39.6 (C-24), 39.4 (C-10), 36.1 (C-22), 35.8 (C-20), 35.2 (C-7), 32.6 (C-1), 30.6 (C-2), 29.7 (C-8), 29.1 (C-16), 27.9 (C-25), 24.0 (C-15), 23.8 (C-23), 21.1 (C-11), 22.7 (Me-26), 22.5 (Me-27), 18.5 (Me-21), 18.2 (Me-19), 12.0 (Me-18).

### 3.5. 3β,6β-Diacetate-5α-cholestan-5α-yl Sodium Sulfate (**3**)

To a stirred solution of 0.5 mg of pure **1** in 0.5 mL of dry pyridine was added 0.3 mL of Ac_2_O. After the mixture was stirred for 12 h at room temperature, evaporation under vacuum gave 0.7 mg of **3** as a white solid: HRESIMS (positive ion mode, CH_3_OH) *m*/*z* 629.3089 ([M + Na]^+^, calcd. for C_31_H_51_SO_8_Na_2_^+^ 629.3100); ^1^H-NMR (C_6_D_6_): δ 0.61 (3H, s, Me-18), 0.94 (6H, d, *J* = 6.6 Hz, Me-26and Me-27), 0.98 (3H, d, *J* = 6.6 Hz, Me-21), 1.14 (3H, s, Me-19), 1.52 (3H, s, COMe), 1.70 (3H, s, COMe), 5.43 (1H, br s, H-6α), 5.72 (1H, m, H-3α); ^13^C-NMR (C_6_D_6_): δ 11.9 (C-18), 17.2 (C-19), 18.5 (C-21), 20.1 (COMe), 20.4 (COMe), 22.4 (C-26 and C-27), 69.9 (C-3), 75.1 (C-6), 168.8 (CO), 169.4 (CO).

### 3.6. Transactivation Experiments

HepG2 cells were plated in a 24-wells plate, at 5 × 10^4^ cells/well, and transfected with 75 ng of pSG5-PXR, 75 ng of pSG5-RXR, 125 ng of pCMV-β-galactosidase, and with 250 ng of the reporter vector pCYP3A4promoter-TKLuc, using Fugene HD transfection reagent (Roche). At 24 h post-transfection, cells were primed with Rifaximin, **1** and **2** (10 μM) or with the combination of Rifaximin (10 μM) plus compounds **1** and **2** (50 μM). After treatments, cells were lysed in 100 μL Lysis Buffer (25 mM TRIS-phosphate pH 7.8; 2 mM DTT; 10% glycerol; 1% Triton X-100) and 20 μL cellular lysate was assayed for Luciferase activity using the Luciferase Assay System (Promega). Luminescence was measured using an automated luminometer (Glomax 20/20, Promega). Luciferase activities were normalized for transfection efficiencies by dividing the Luciferase relative light units (RLU) by β-galactosidase activity (βgal) expressed from cells co-transfected with pCMVβgal. All experiments were performed in triplicate.

### 3.7. Cells Culture, RNA Extraction and Real-Time PCR

HepG2 cells were maintained at 37 °C in E-MEM supplemented with 10% FBS, 1% l-glutamine and 1% penicillin/streptomycin. To evaluate PXR target genes expression, serum starved HepG2 cells were stimulated for 18 h with Rifaximin and compound **1** and **2** (10 μM). Total RNA was extracted using the TRIzol reagent (Invitrogen), purified of the genomic DNA by DNAase I treatment (Invitrogen) and random reverse-transcribed with Superscript II (Invitrogen). 10 ng template was amplified using the following reagents: 0.2 μM of each primer and 10 μL of KAPA SYBR FAST Universal qPCR Kit (KAPA BIOSYSTEMS). All reactions were performed in triplicate and the thermal cycling conditions were: 3 min at 95 °C, followed by 40 cycles of 95 °C for 15 s, 58 °C for 20 s and 72 °C for 30 s. The relative mRNA expression was calculated and expressed as 2^−(ΔΔCt)^. Primers used for qRT-PCR were:

hGAPDH: GAAGGTGAAGGTCGGAGT and CATGGGTGGAATCATATTGGAA;
hCYP3A4: CAAGACCCCTTTGTGGAAAA and CGAGGCGACTTTCTTTCATC;
hMDR1: GTGGGGCAAGTCAGTTCATT and TCTTCACCTCCAGGCTCAGT.

### 3.8. Statistical Analysis

All values are expressed as means ± standard error (SE) of n observations/group. Comparisons of two groups were made with a one-wayANOVA with post hoc Tukey’s test. Differences were considered statistically significant at values of *P* < 0.05.

### 3.9. Computational Details

Molecular docking of phallusiasterol A in the three-dimensional X-ray structures of the PXR LBD (PDB codes: 3hvl, 1nrl and 1m13) without the co-crystallized inhibitor and waters were carried out using the AutoDock software package (version 4.2, The Scripps Research Institute, La Jolla, CA, USA) [23]. Ligands and receptor structures were converted to AutoDock format files using the ADT software (The Scripps Research Institute, La Jolla, CA, USA) and the Gesteiger-Marsili partial charges were then assigned. A box around the binding pocket has defined the docking area and grids points of 48 × 40 × 38 with 0.375 Å spacing were calculated within the this area for all the ligand atom types using AutoGrid4. For each ligand, 100 separate docking calculations were performed. Each docking run consisted of 25 million energy evaluations using the Lamarckian genetic algorithm local search (GALS) method. Otherwise default docking parameters were applied. The docking conformations were clustered on the basis of the root-mean square deviation values (rmsd tolerance = 1.5 Å) between the cartesian coordinates of the ligand atoms and were ranked based on the AutoDock scoring function.

## 4. Conclusions

Phallusiasterols A (1) and B (2) to our knowledge are the first sulfated sterols isolated from tunicates. These unusual marine steroids have been recognized as new potential nuclear receptor ligands and their possible role in regulating the PXR activity has been investigated. *In vitro* assays demonstrated that, in spite of their close structure similarity (phallusiasterol B is the C-6 epimer of phallusiasterol A), only phallusiasterol A is endowed with PXR agonistic activity, indicating a crucial role in the ligand-receptor binding of phallusiasterols of the configuration at C-6. The activity of 1 is comparable to that of rifaximin, a well characterized ligand for the human PXR. Supporting the role of phallusiasterol A as PXR regulator, we have observed that it effectively increases the expression of two PXR target genes, CYP3A4 and MDR1, in a human hepatocyte cell line, whereas 2 failed to induce them. Both compounds 1 and 2 were not PXR antagonists. The experimental biological results have been also validated by docking studies which allowed rationalization of the activity of phallusiasterol A. 

## Supplementary Files

Supplementary File 1 Supplementary Information (PDF, 1416 KB)
