# Supplementary material for: Phallusiasterols A and B: Two New Sulfated Sterols from the Mediterranean Tunicate Phallusia fumigata and Their Effects as Modulators of the PXR Receptor
Source: Mar Drugs. 2014 Apr 3;12(4):2066–78. doi: 10.3390/md12042066 (PMC4012412; doi:10.3390/md12042066)
Supplement: Supplementary File 1 — Supplementary Information (PDF, 1416 KB) [file marinedrugs-12-02066-s001.pdf]

Supplementary Information

Figure S1. ^{1}H-NMR spectrum of phallusiasterol A (**1**) (C_5D_5N, 700 MHz).

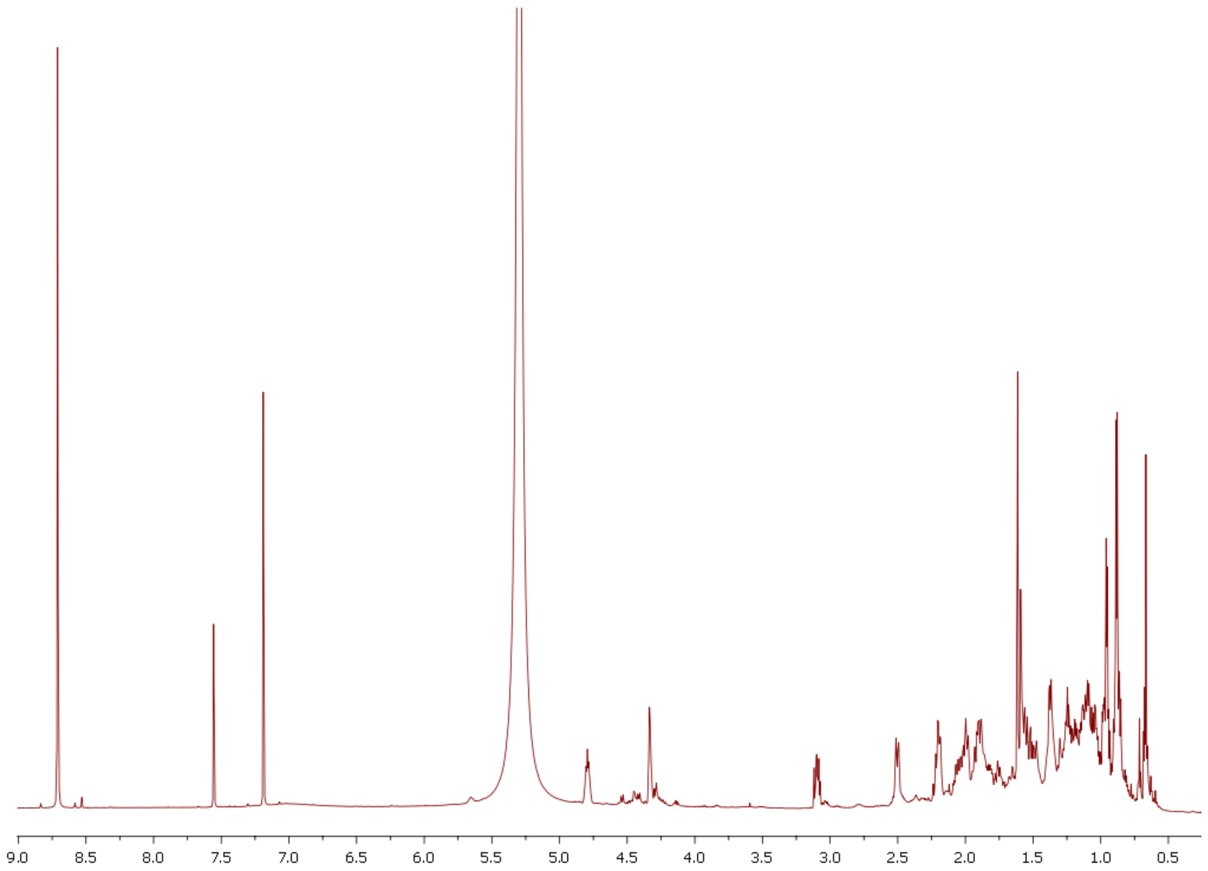

Figure S2. ^{13}C-NMR spectrum of phallusiasterol A (1) (C_5D_5N, 700 MHz).

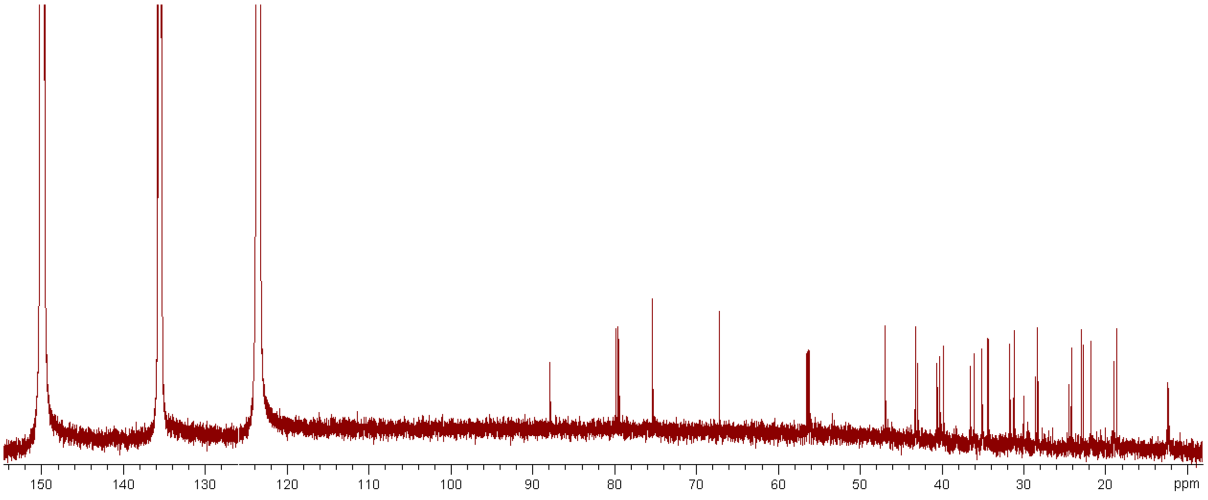

Figure S3. COSY spectrum of phallusiasterol A (**1**) (C_5D_5N, 700 MHz).

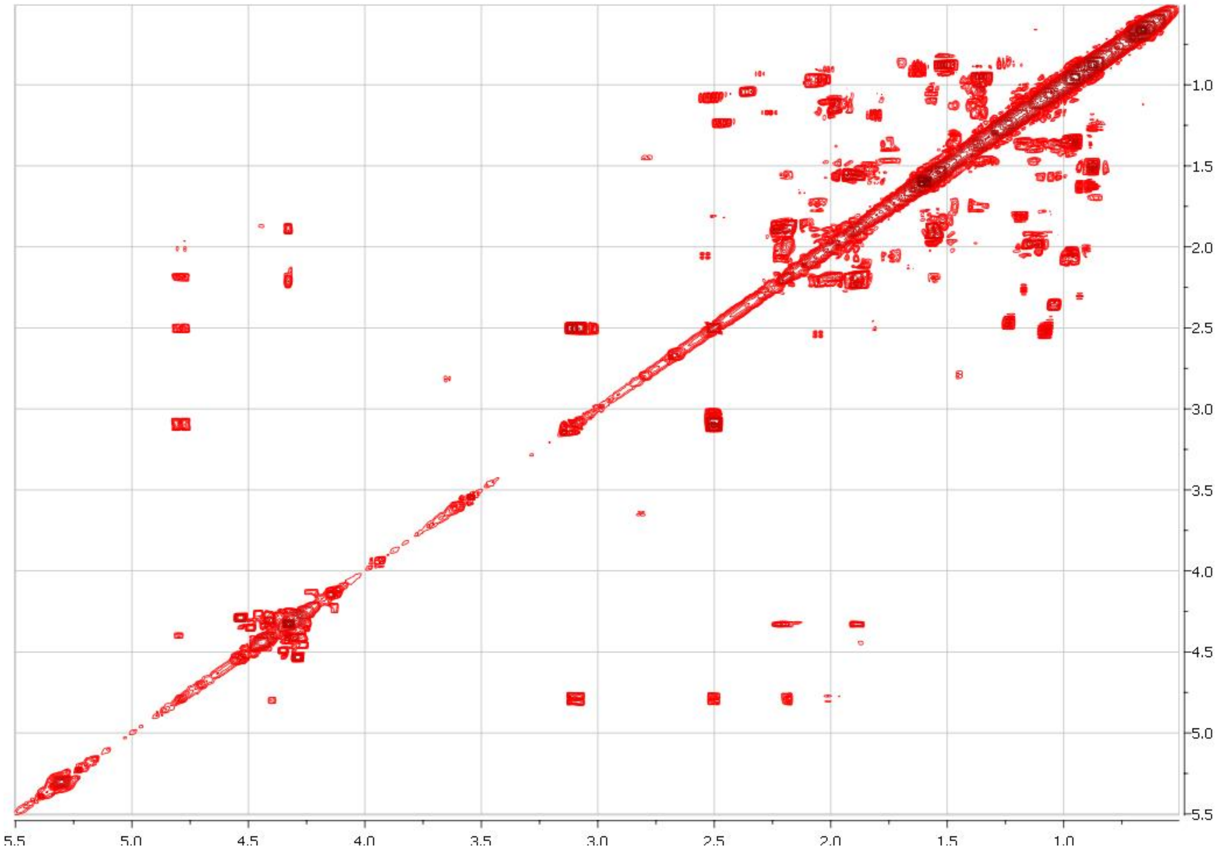

Figure S4. HSQC spectrum of phallusiasterol A (**1**) (C_5D_5N, 700 MHz).

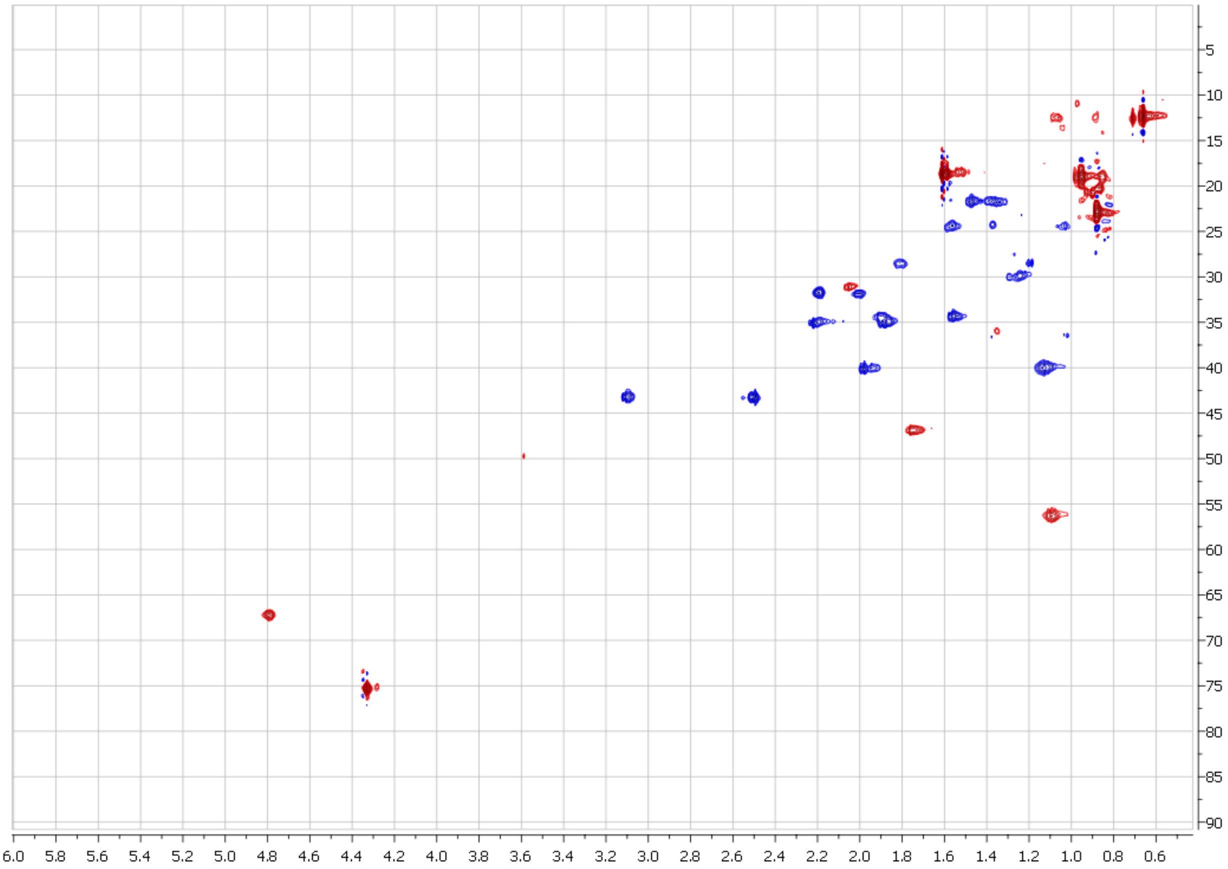

Figure S5. HMBC spectrum of phallusiasterol A (**1**) (C_5D_5N, 700 MHz).

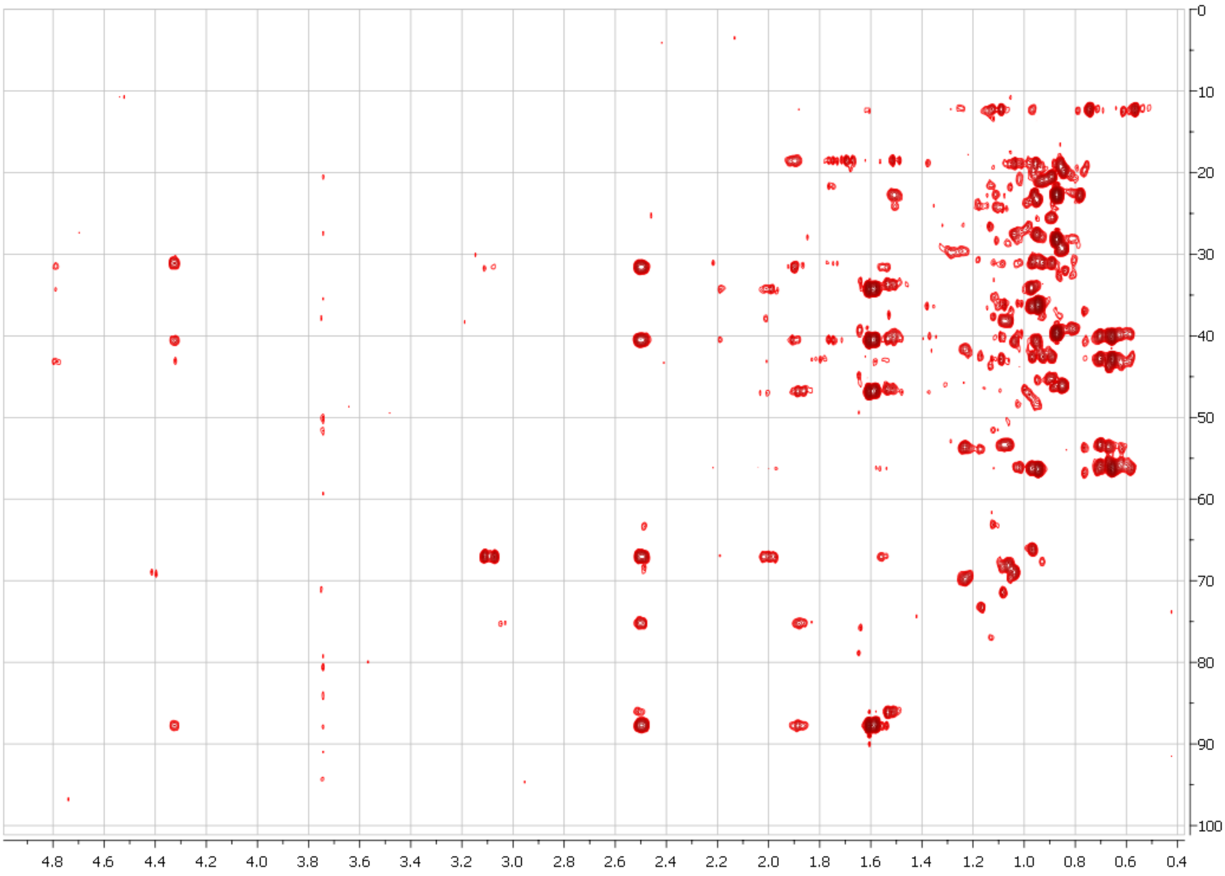

Figure S6. ROESY spectrum of phallusiasterol A (**1**) (C_5D_5N, 700 MHz).

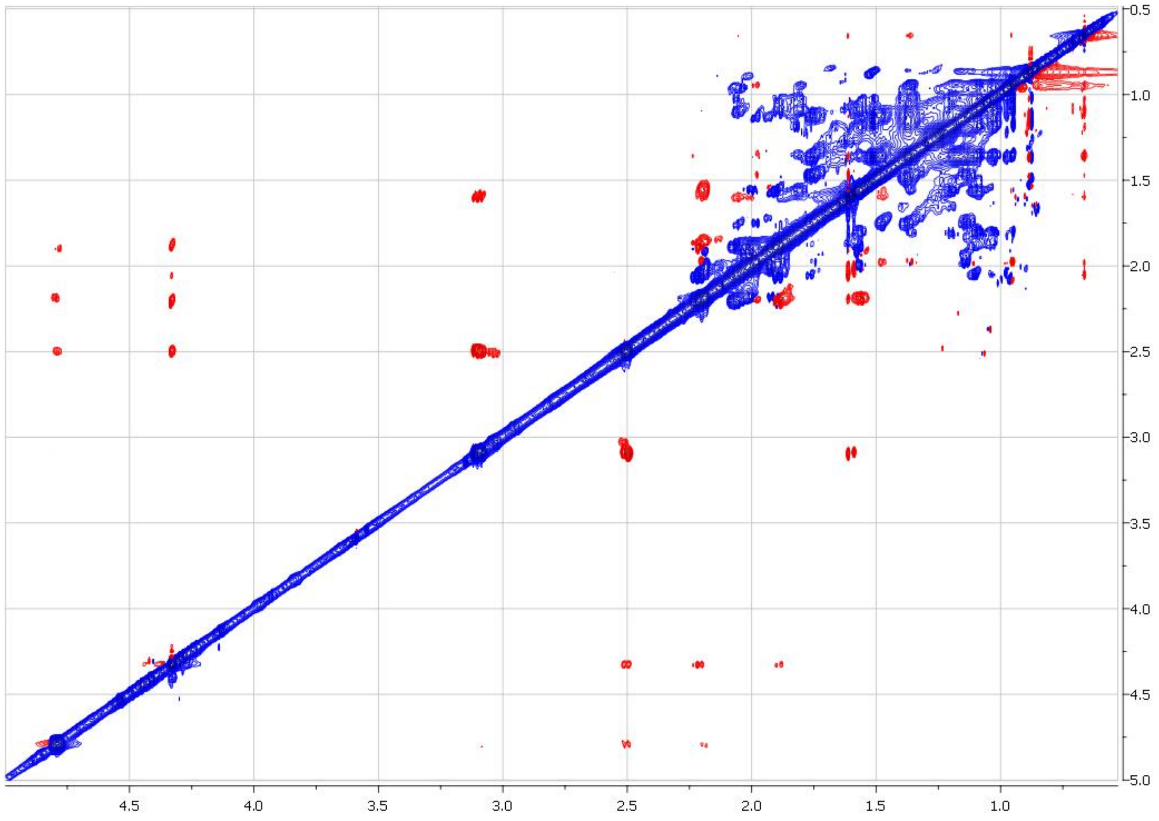

Figure S7. Positive-ion HRESI MS spectrum of phallusiasterol A (**1**).

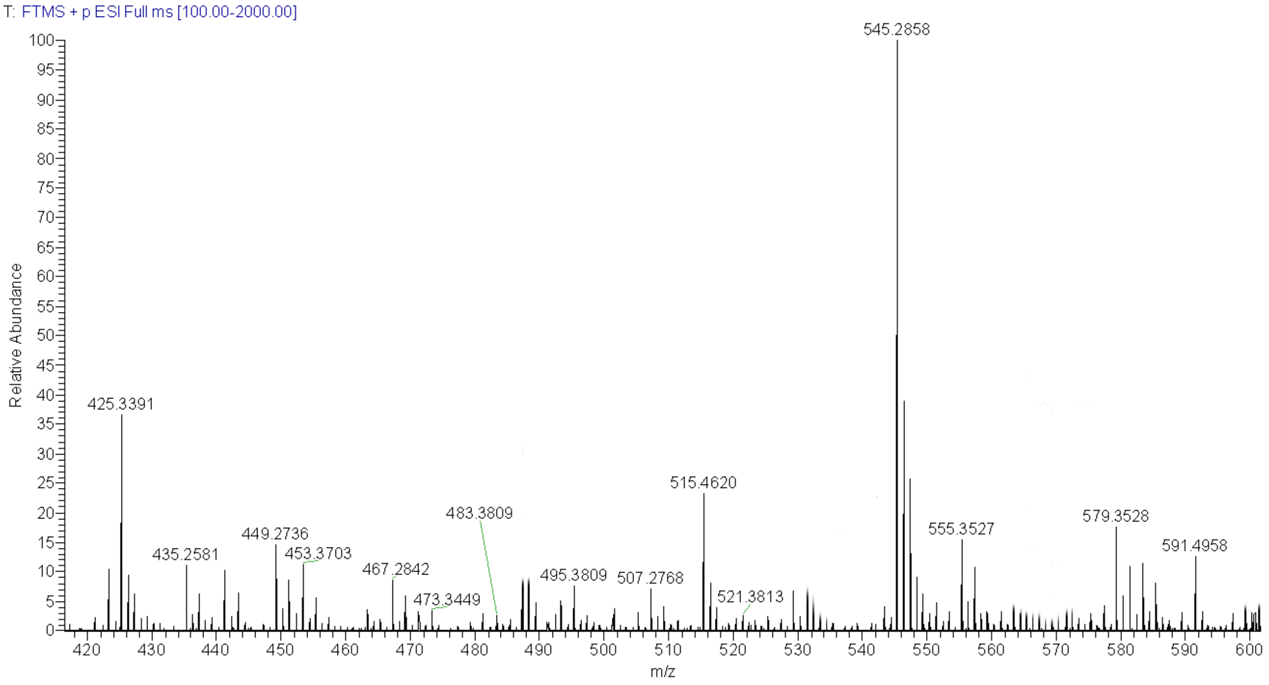

Figure S8. Positive-ion HRESI MS spectrum of phallusiasterol B (**2**).

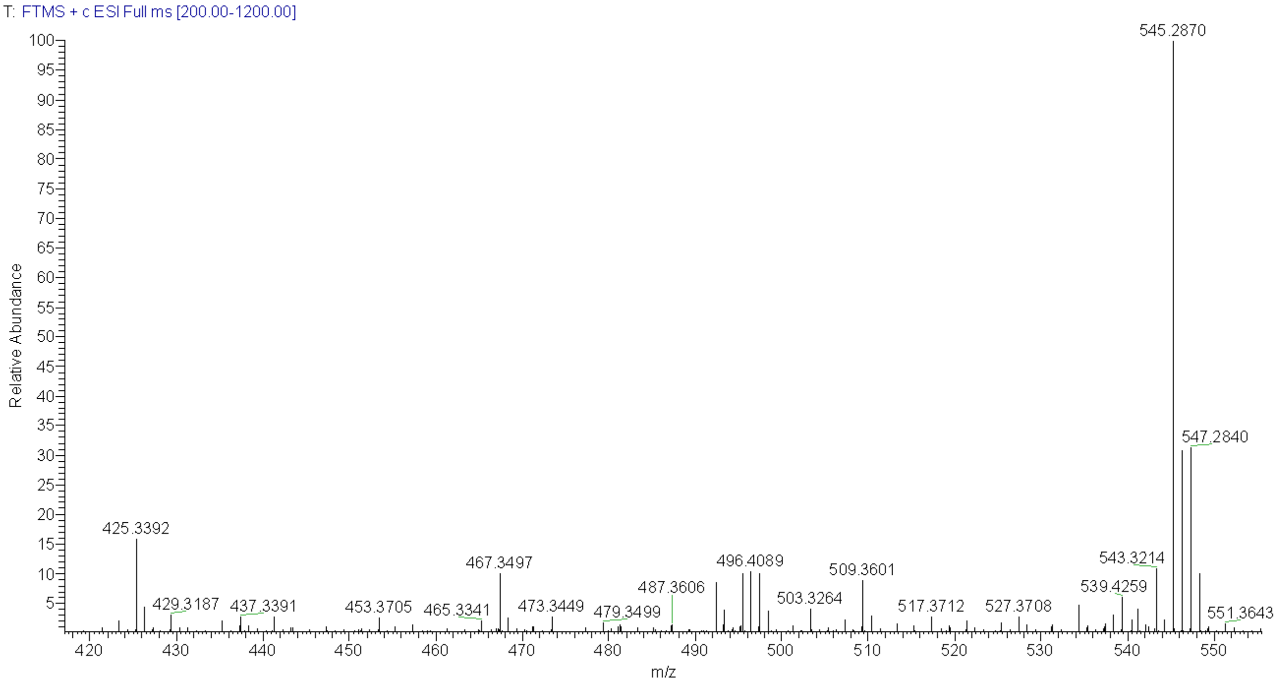

Figure S9. ^{1}H-NMR spectrum of phallusiasterol B (**2**) (C_5D_5N, 700 MHz).

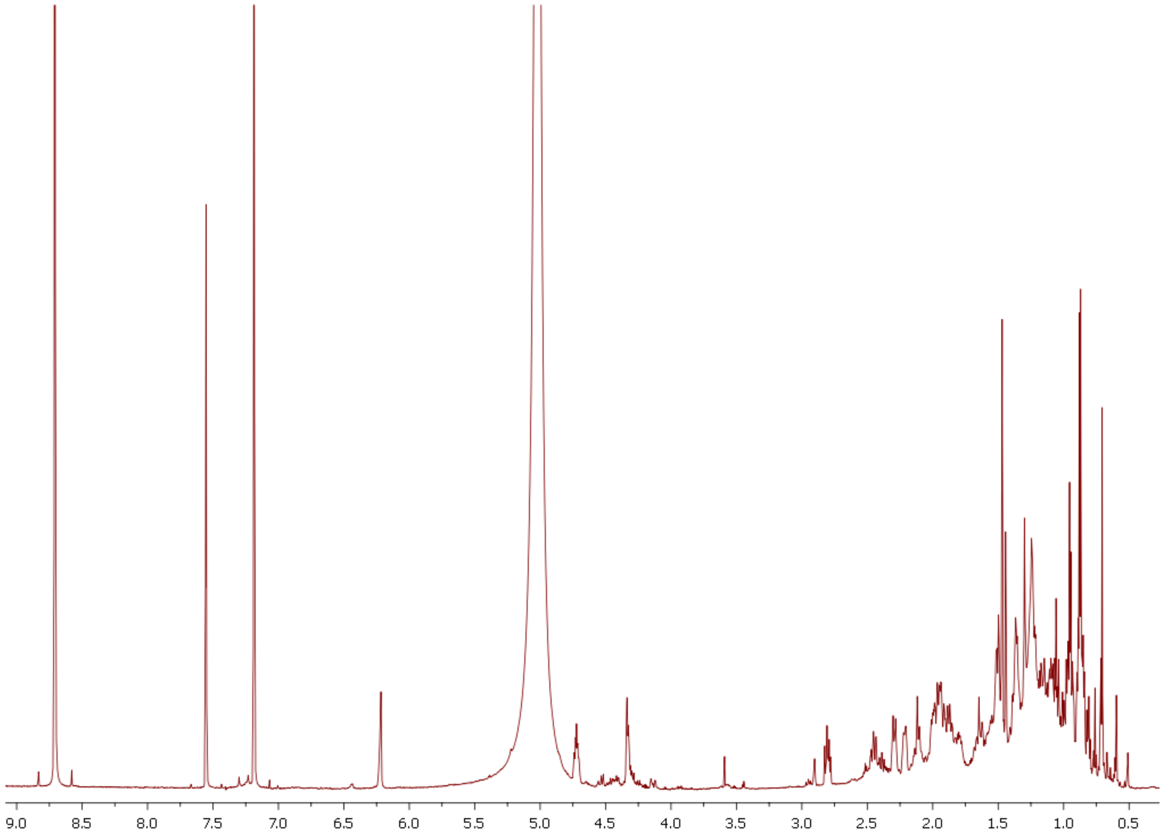

Figure S10. COSY spectrum of phallusiasterol B (**2**) (C_5D_5N, 700 MHz).

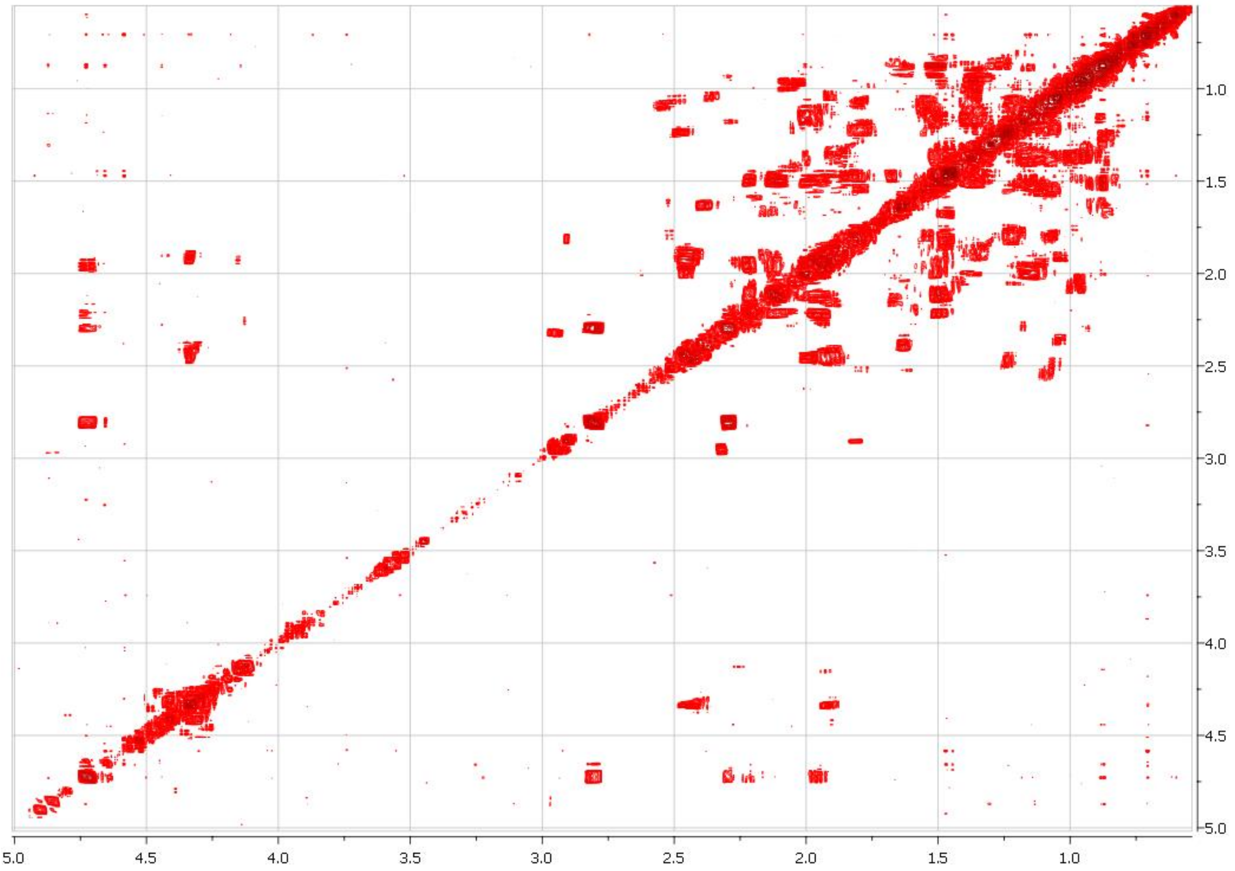

Figure S11. HSQC spectrum of phallusiasterol B (**2**) (C_5D_5N, 700 MHz).

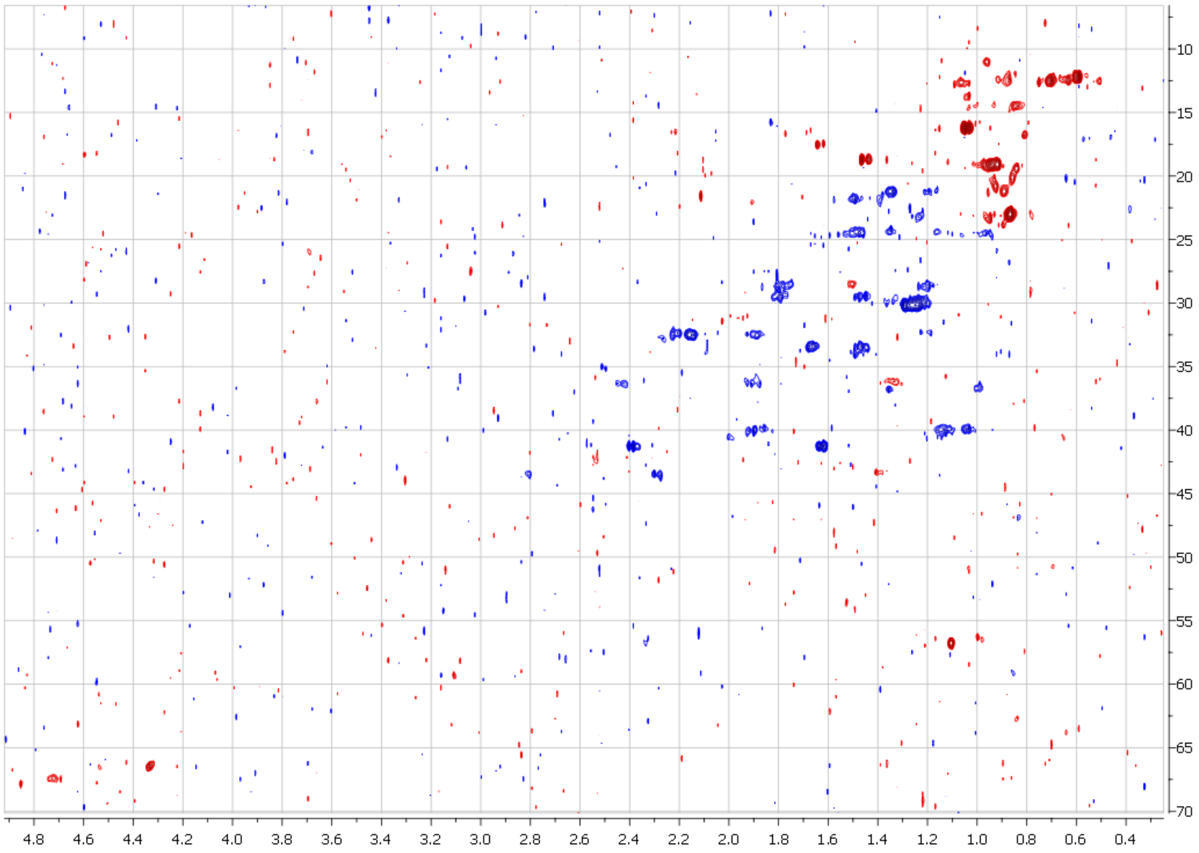

Figure S12. HMBC spectrum of phallusiasterol B (**2**) (C_5D_5N, 700 MHz).

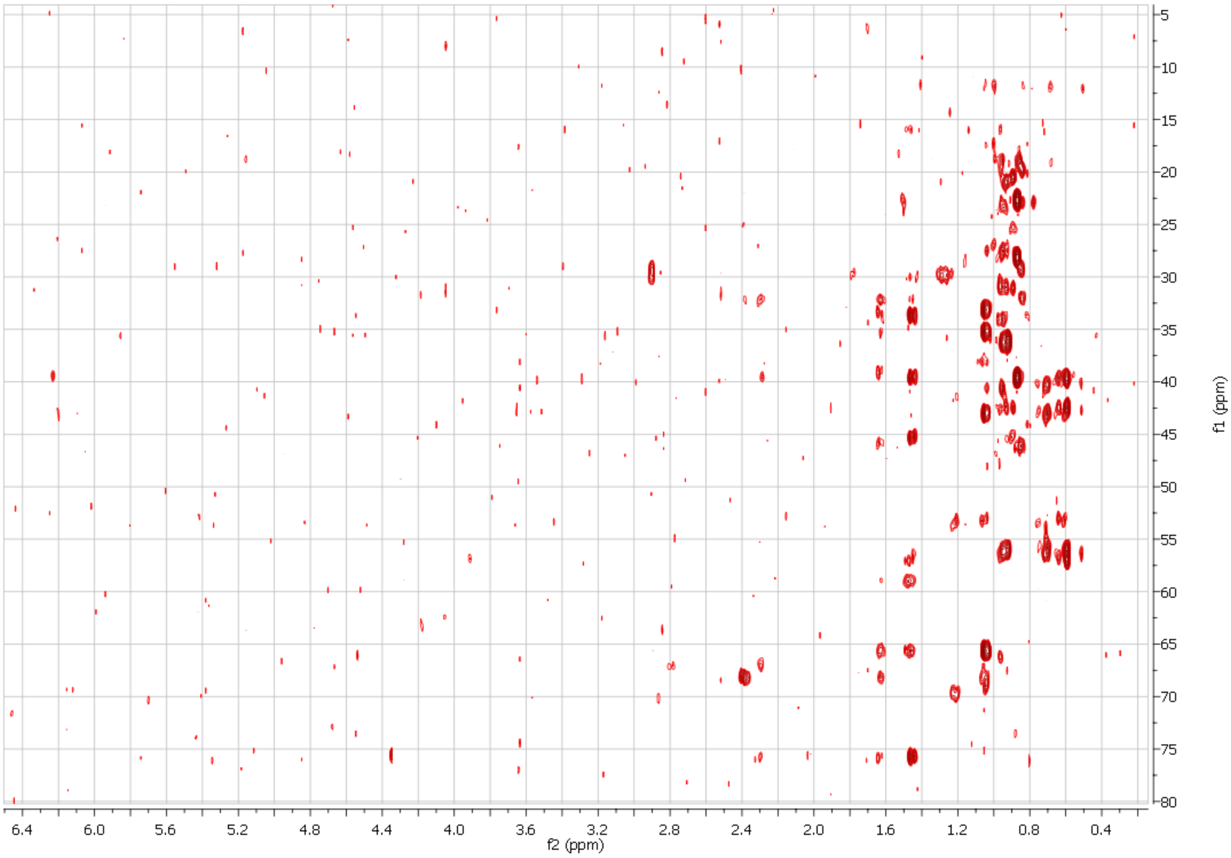

Figure S13. TOCSY spectrum of phallusiasterol B (**2**) (C_5D_5N, 700 MHz).

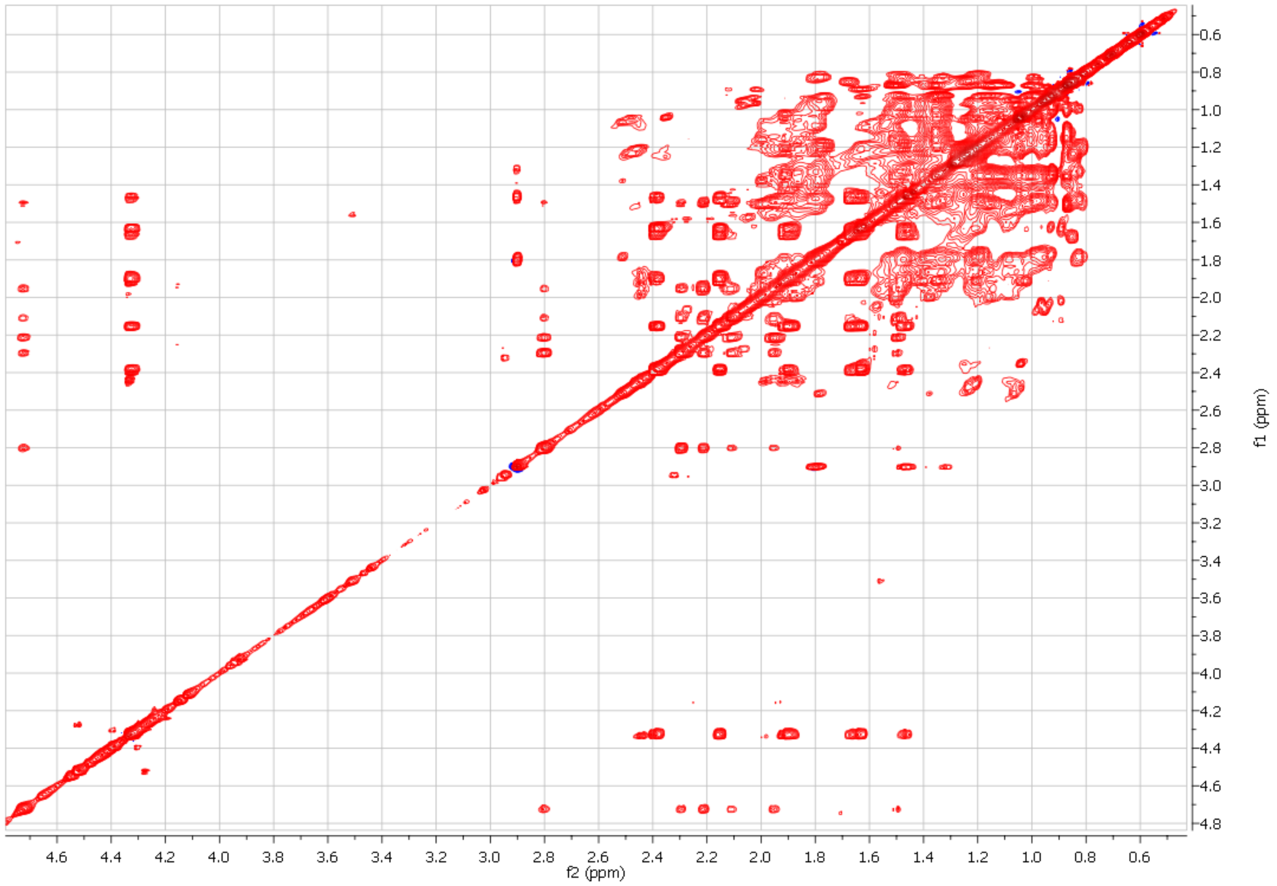

Figure S14. ROESY spectrum of phallusiasterol B (**2**) (C_5D_5N, 700 MHz)

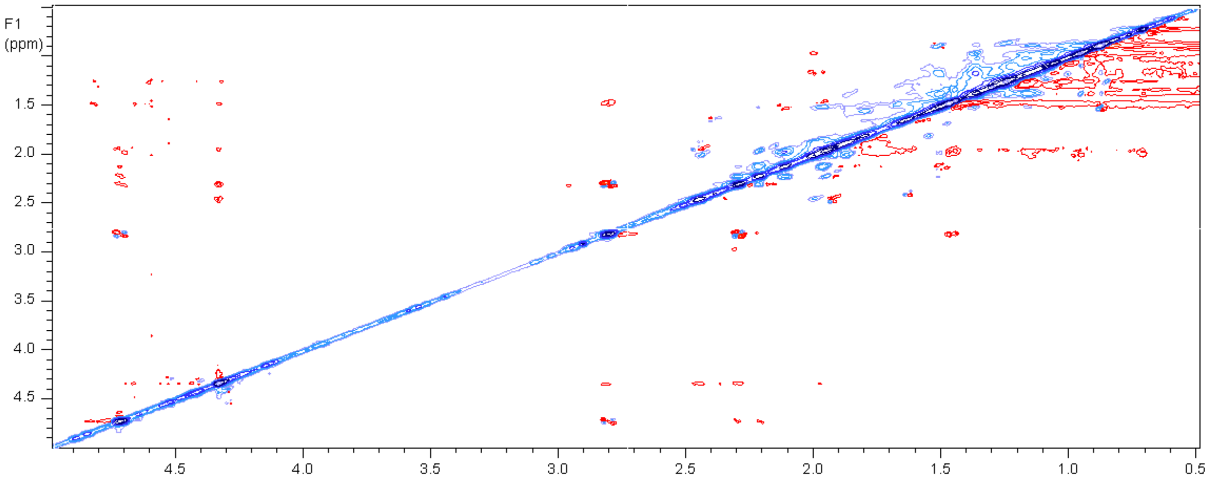

Figure S15. ^{1}H-NMR spectrum of phallusiasterol A (**1**) ($CDCl_3$, 700 MHz).

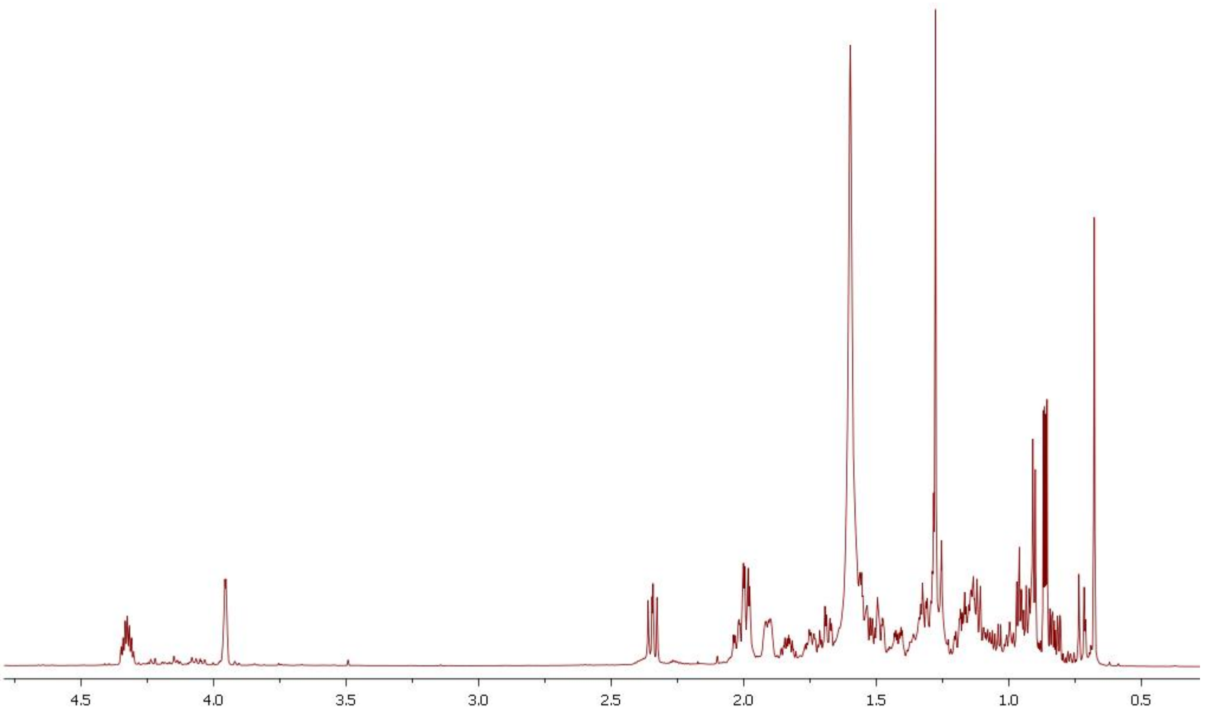

Figure S16. ^{13}C-NMR spectrum of phallusiasterol A (**1**) ($CDCl_3$, 700 MHz).

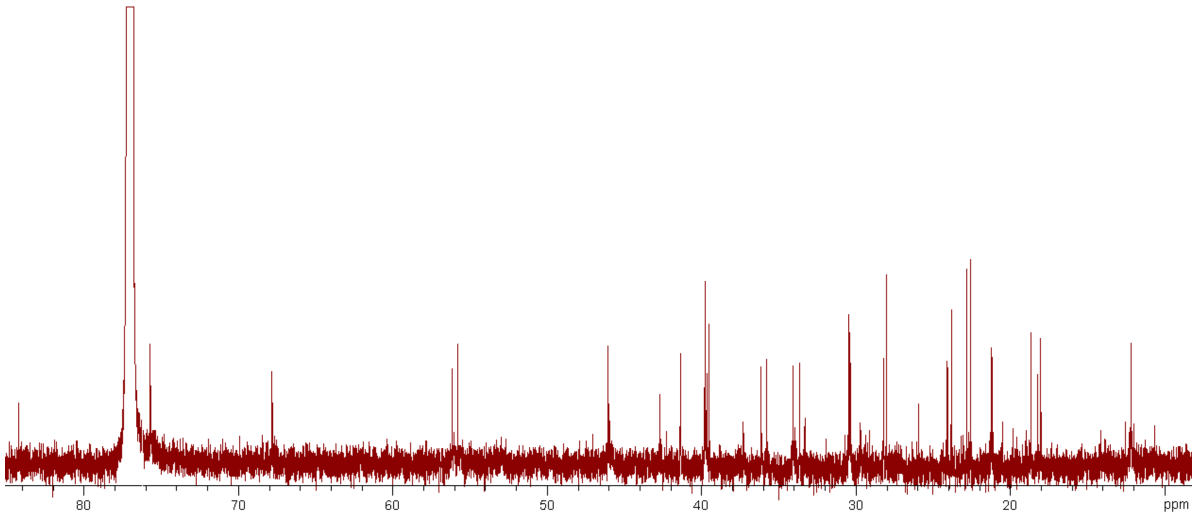

Figure S17. COSY spectrum of phallusiasterol A (**1**) ($CDCl_3$, 700 MHz).

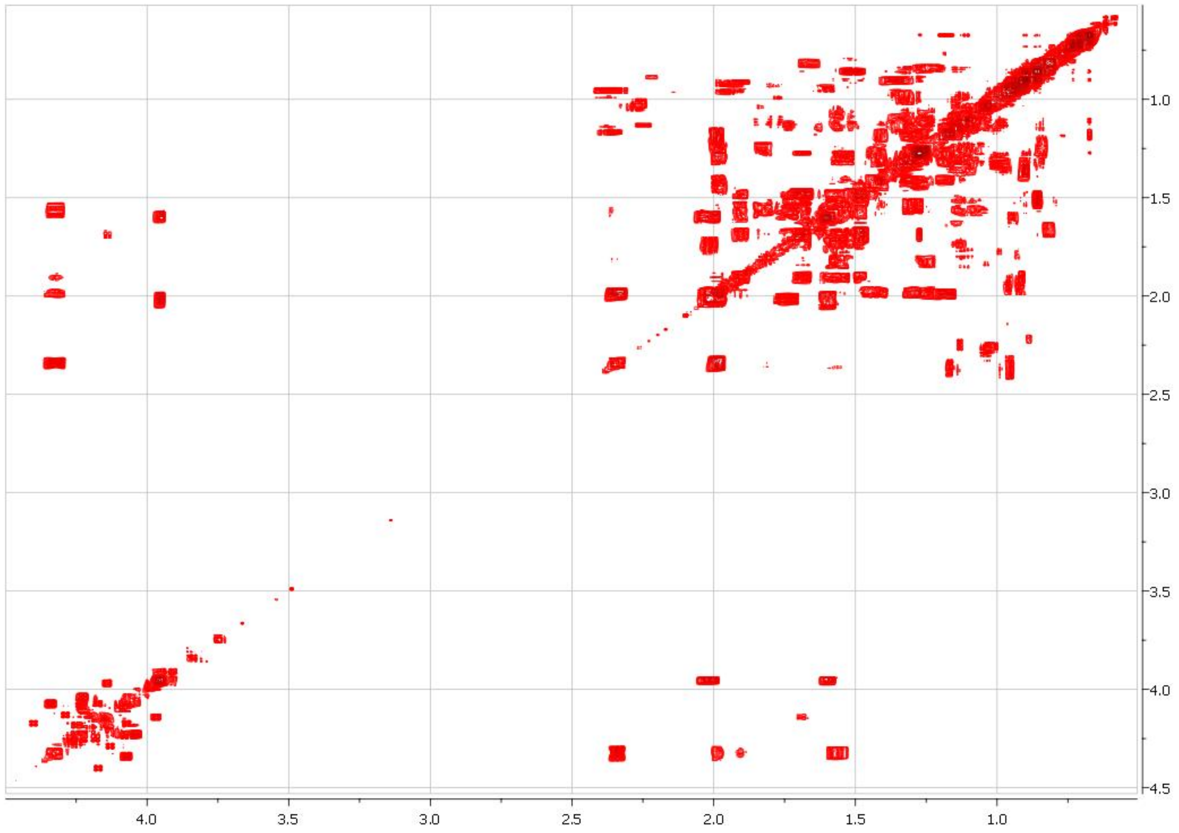

Figure S18. HSQC spectrum of phallusiasterol A (**1**) ($CDCl_3$, 700 MHz).

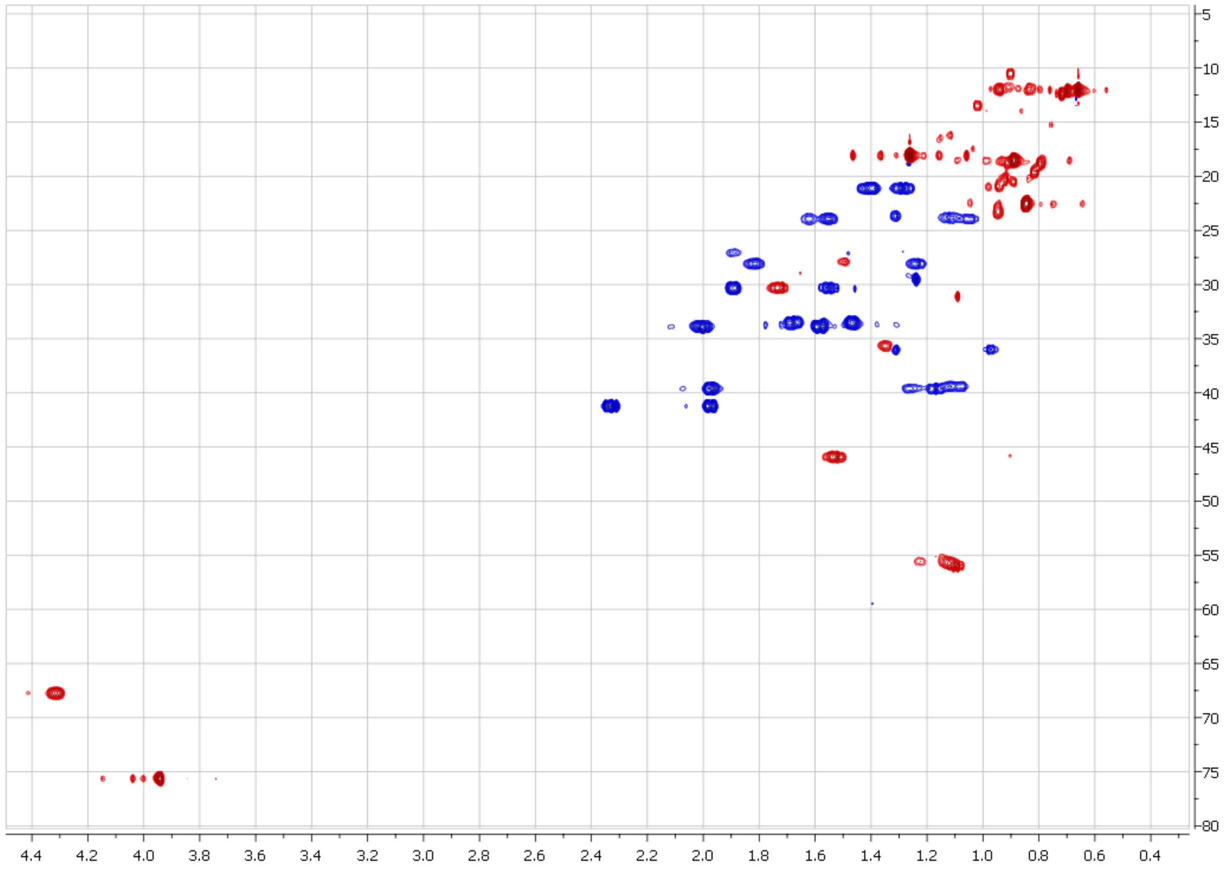

Figure S19. HMBC spectrum of phallusiasterol A (**1**) ($CDCl_3$, 700 MHz).

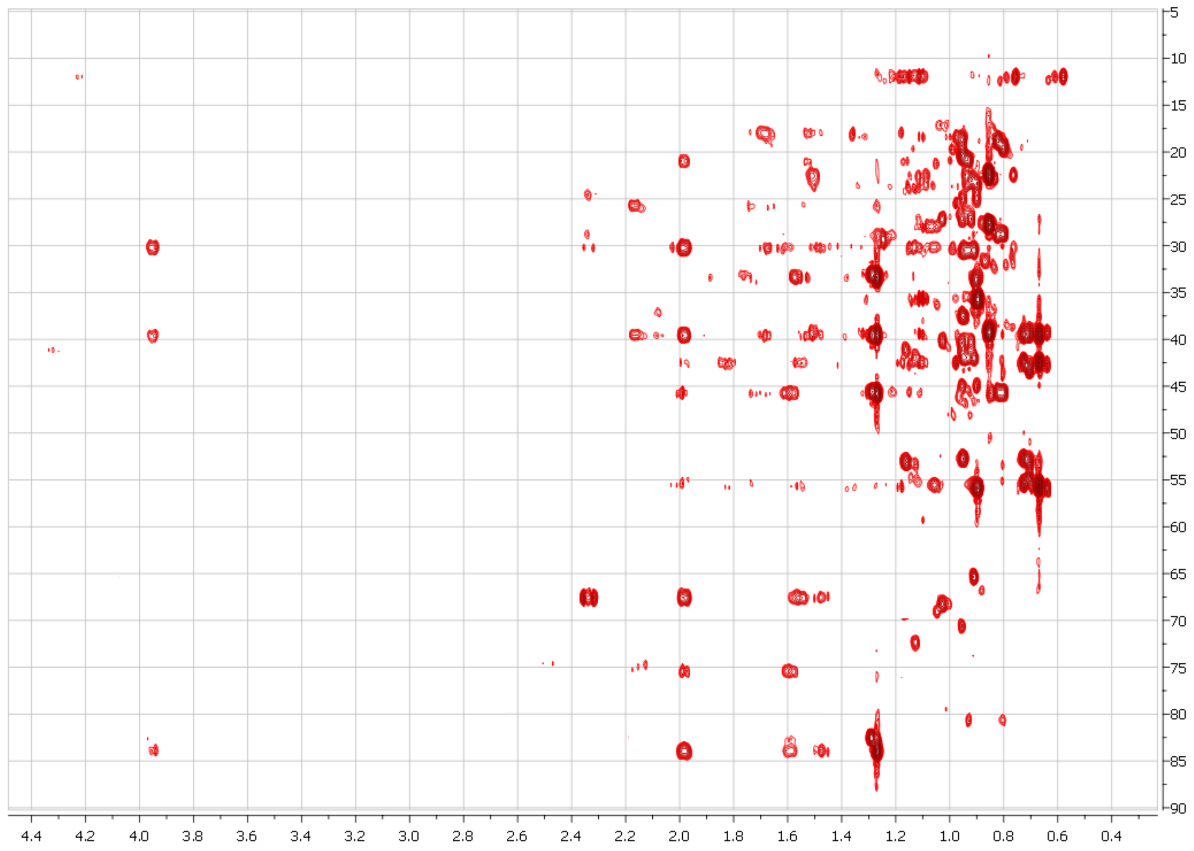

Figure S20. ROESY spectrum of phallusiasterol A (**1**) ($CDCl_3$, 700 MHz).

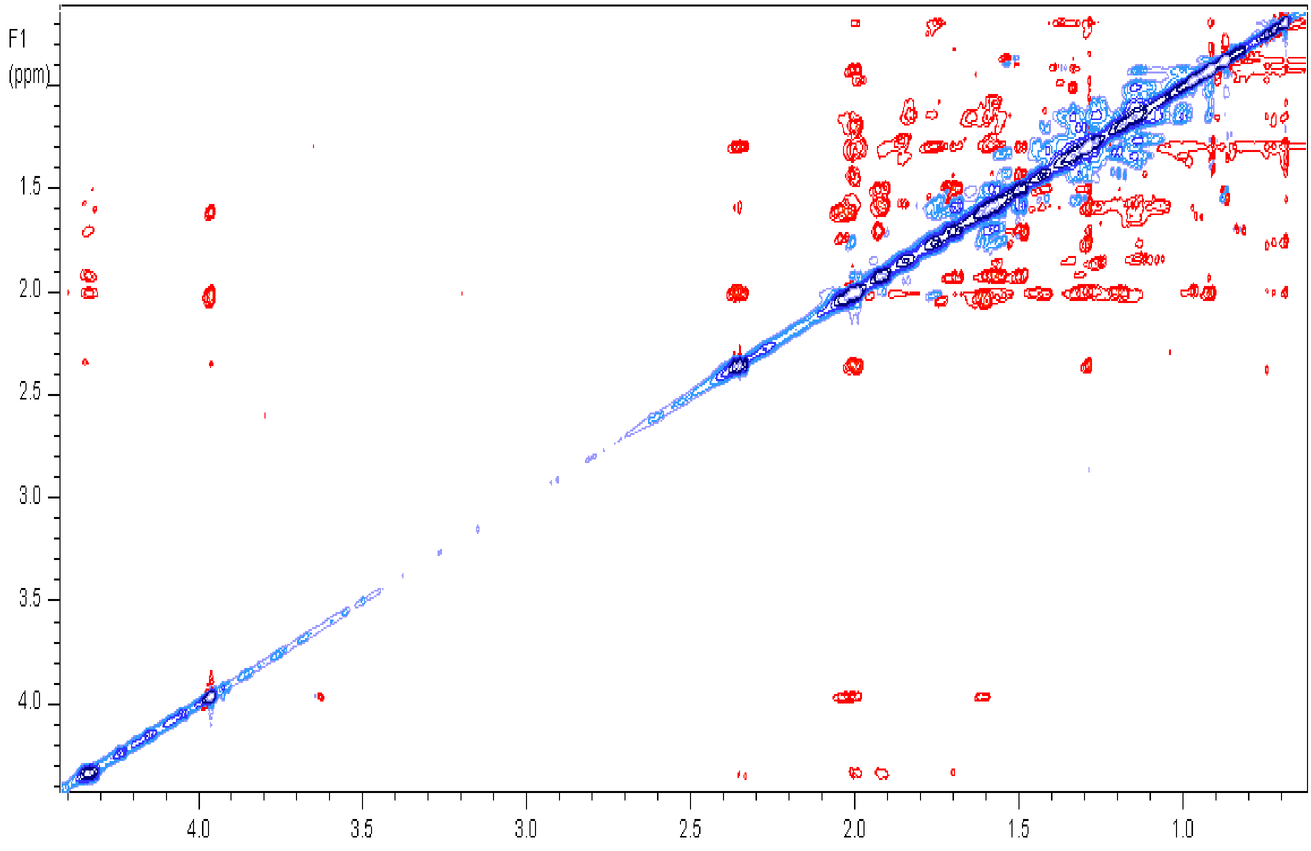

Figure S21. TOCSY spectrum of phallusiasterol A (**1**) ($CDCl_3$, 700 MHz).

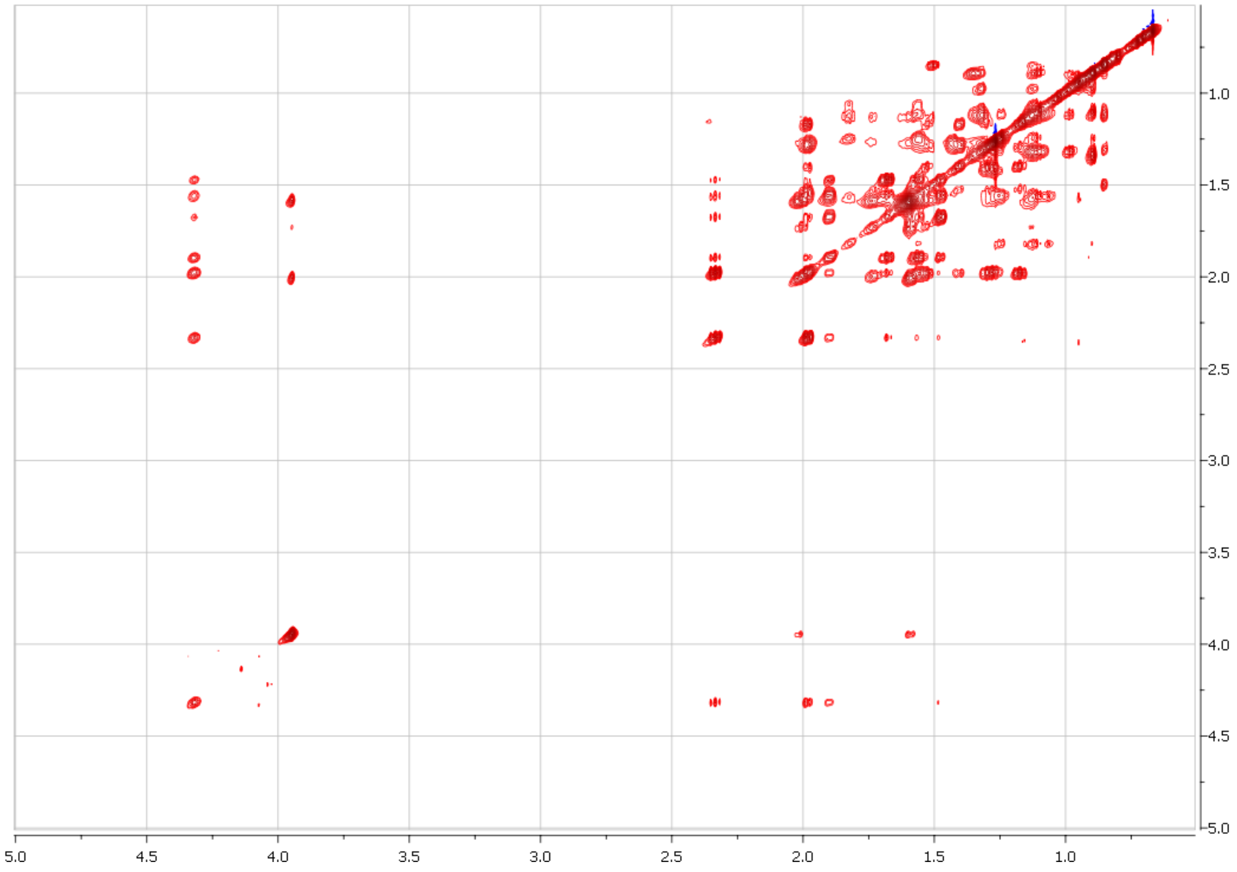

Figure S22. ^{1}H-NMR spectrum of phallusiasterol B (**2**) ($CDCl_3$, 500 MHz).

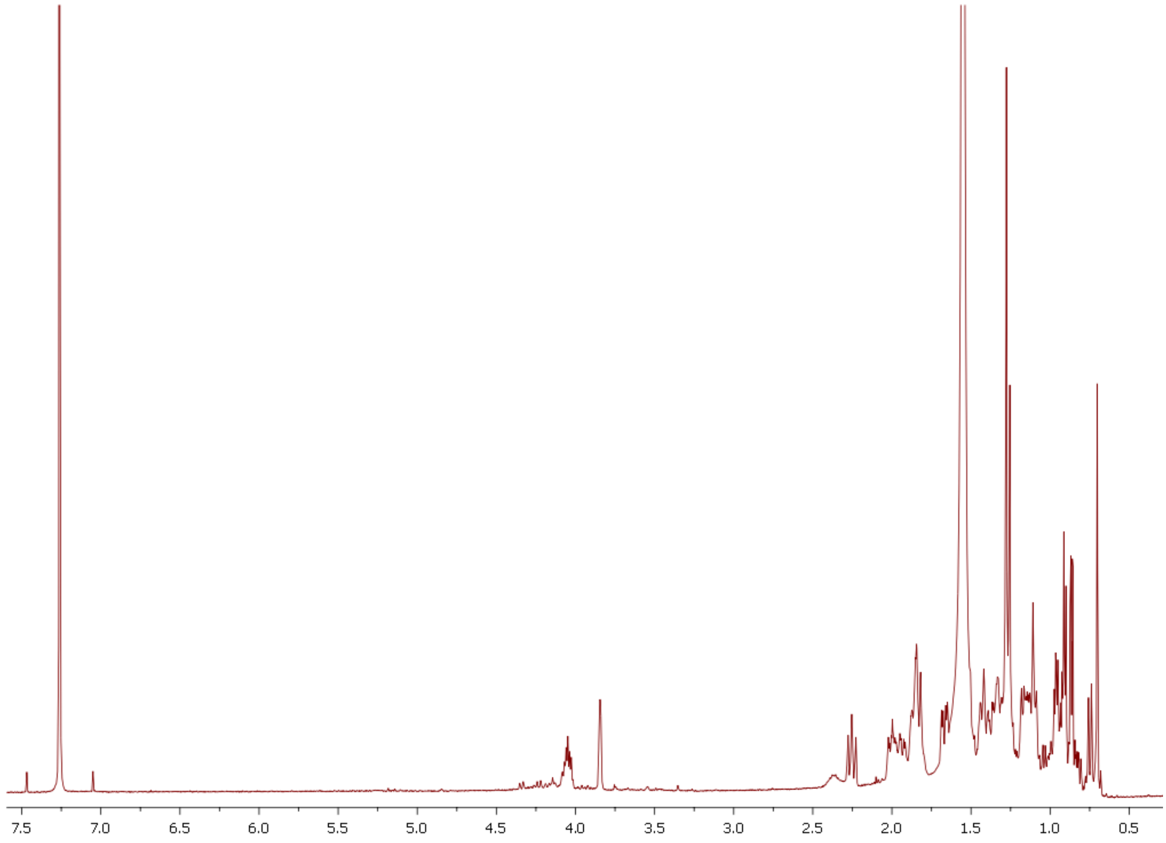

Figure S23. COSY spectrum of phallusiasterol B (**2**) ($CDCl_3$, 500 MHz).

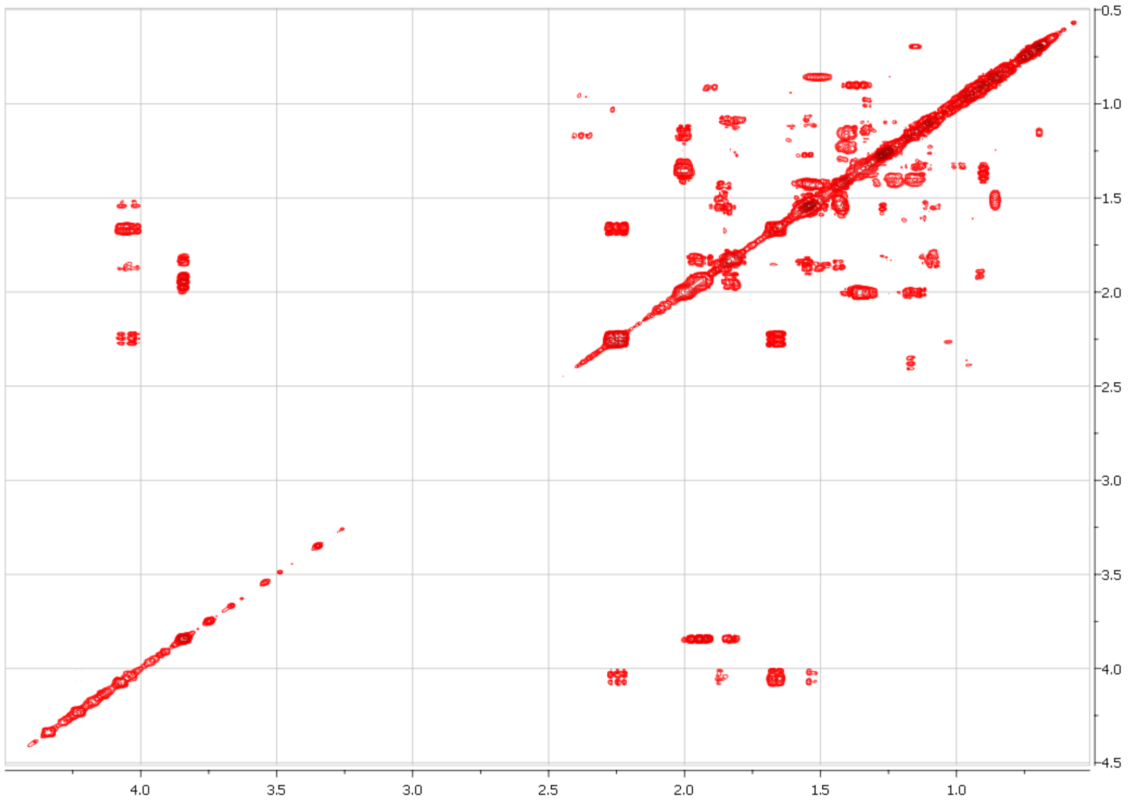

Figure S24. ^{1}H-NMR spectrum of compound **3** (C_6D_6, 700 MHz).

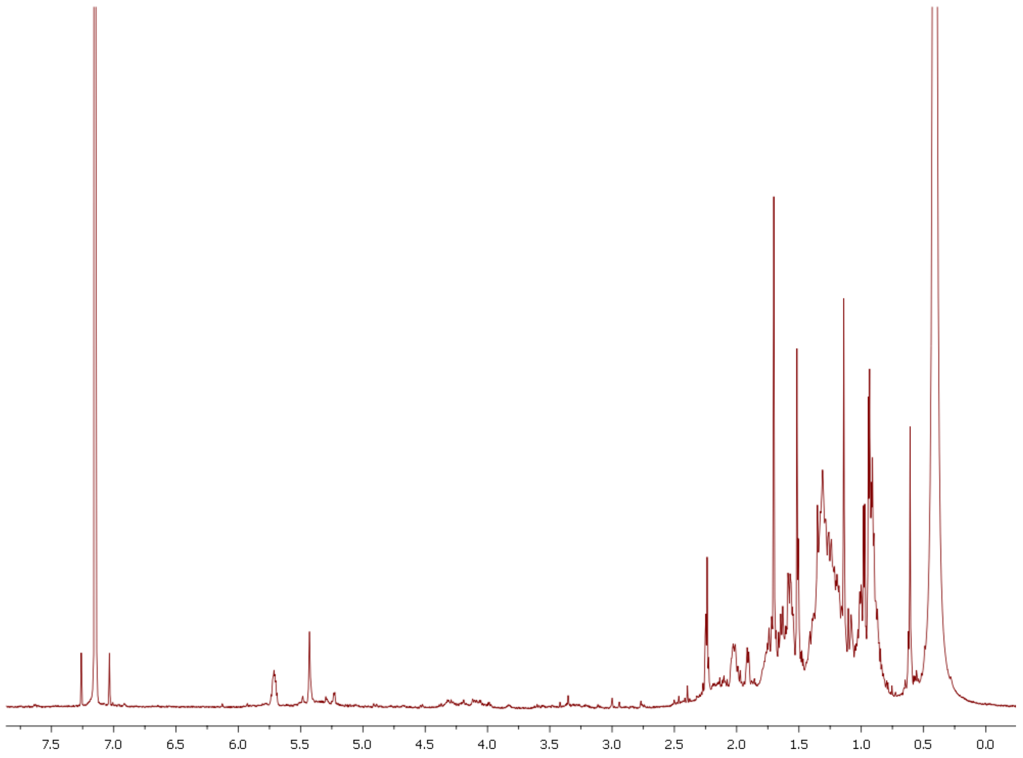

© 2014 by the authors; licensee MDPI, Basel, Switzerland. This article is an open access article distributed under the terms and conditions of the Creative Commons Attribution license (http://creativecommons.org/licenses/by/3.0/).
